# Supplementary figures and images for: Spatio-temporal clusters and patterns of spread of dengue, chikungunya, and Zika in Colombia
Source: PLoS Negl Trop Dis. 2022 Aug 23;16(8):e0010334. doi: 10.1371/journal.pntd.0010334 (PMC9439233; doi:10.1371/journal.pntd.0010334)

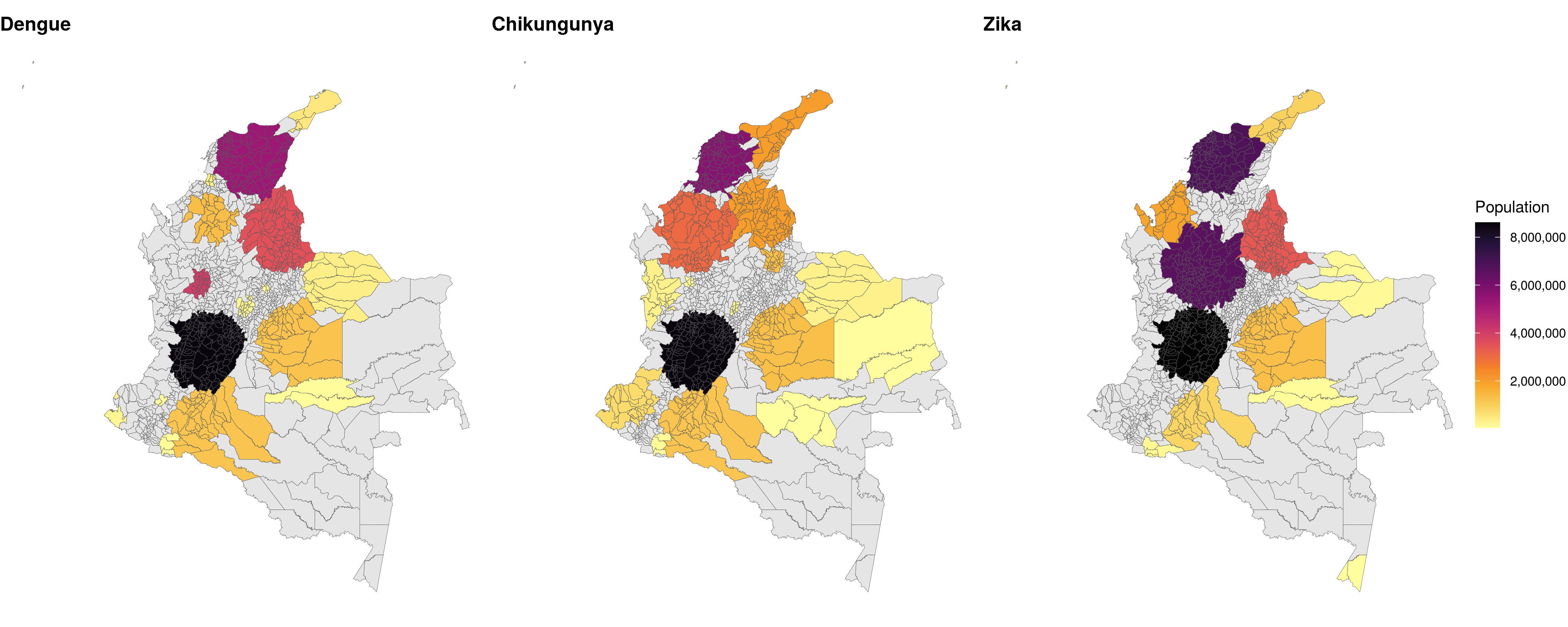

Supplement: S1 Fig — Base layer source: GADM (https://gadm.org/), available at https://www.diva-gis.org/gdata. (PNG) [file pntd.0010334.s001.png]

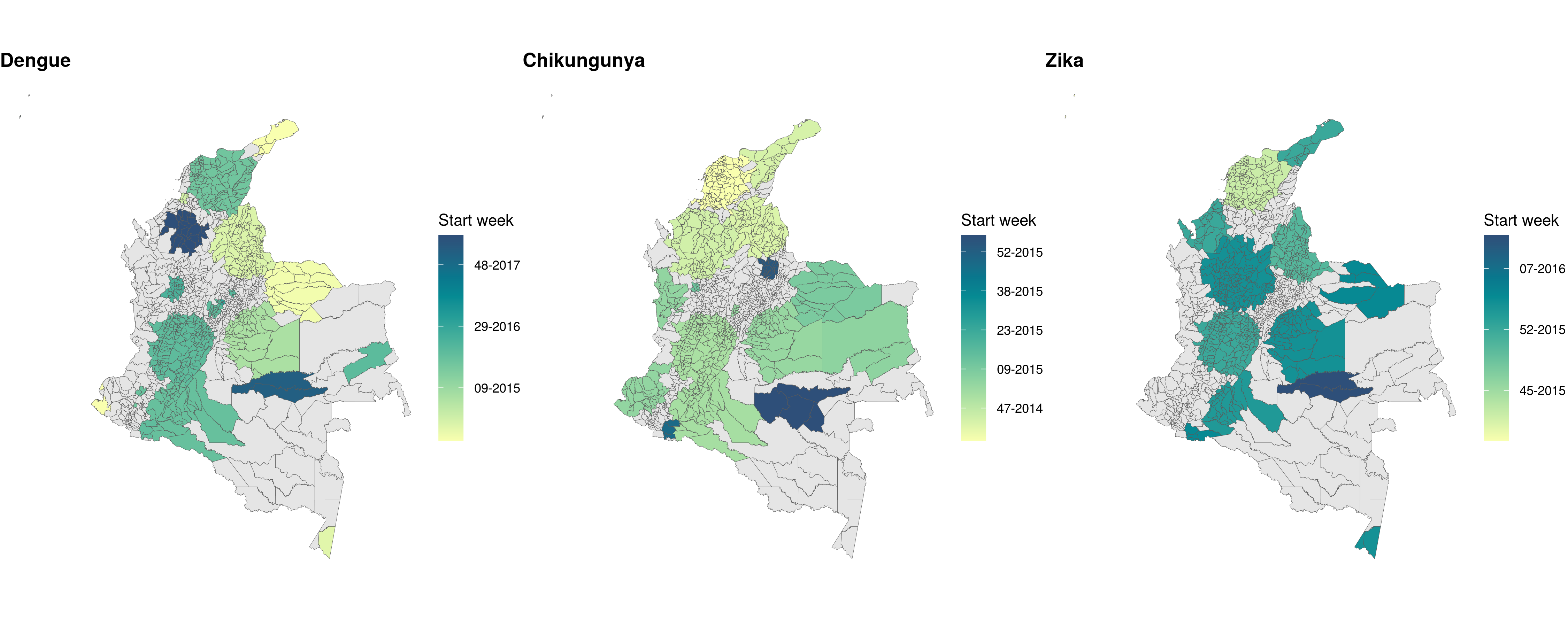

Supplement: S2 Fig — Base layer source: GADM (https://gadm.org/), available at https://www.diva-gis.org/gdata. (PNG) [file pntd.0010334.s002.png]

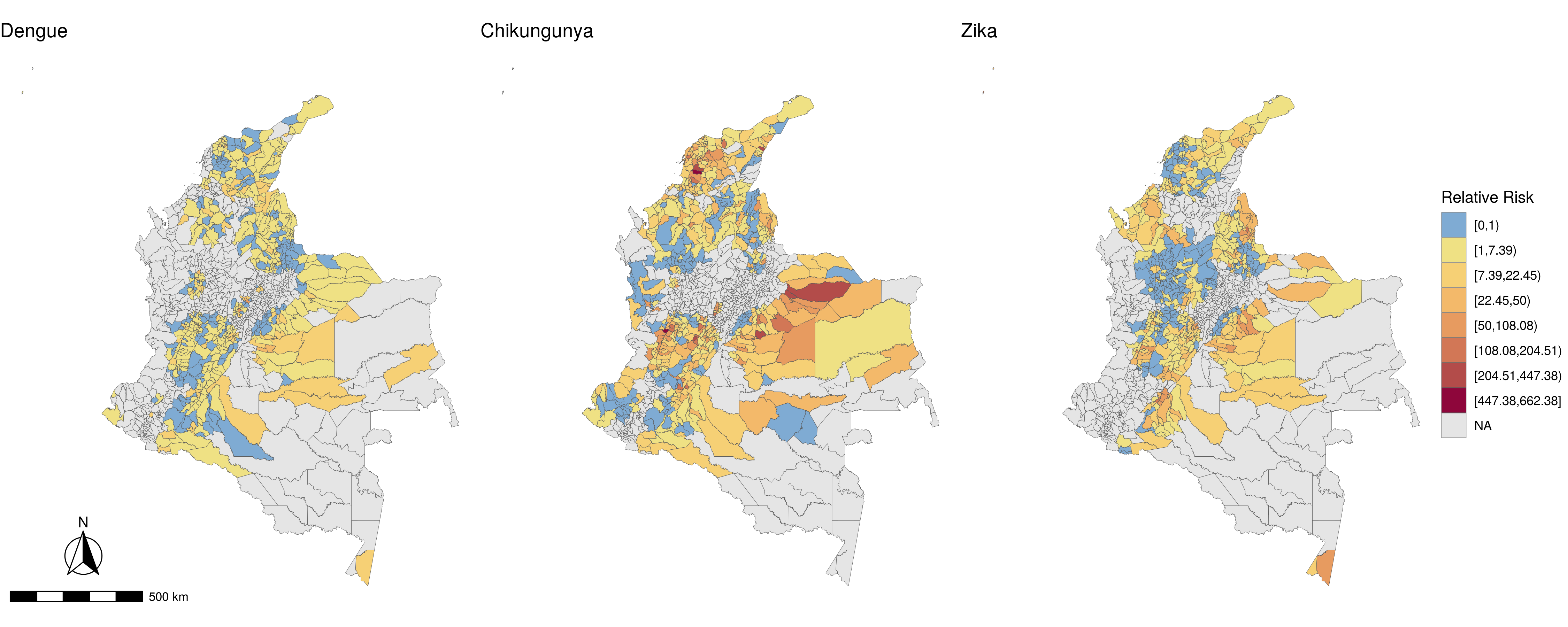

Supplement: S3 Fig — Base layer source: GADM (https://gadm.org/), available at https://www.diva-gis.org/gdata. (PNG) [file pntd.0010334.s003.png]
