# Supplementary material for: Spatio-temporal clusters and patterns of spread of dengue, chikungunya, and Zika in Colombia
Source: PLoS Negl Trop Dis. 2022 Aug 23;16(8):e0010334. doi: 10.1371/journal.pntd.0010334 (PMC9439233; doi:10.1371/journal.pntd.0010334)
Supplement: S1 Appendix — (PDF) [file pntd.0010334.s004.pdf]

S1 Appendix
Case definitions for dengue, chikungunya and Zika

Case definitions for probable and confirmed cases of dengue, chikungunya and Zika following the official protocols of the National Institute of Health of Colombia [1-3].

**DENGUE [1]**	
**Probable case**	Patient from an endemic area who meets the definition of dengue with or without warning signs.<br><br>**Dengue without warning signs**<br>Acute febrile illness of 2 to 7 days of evolution in which two or more of the following manifestations are observed: headache, retro-ocular pain, myalgia, arthralgia, skin eruption, rash or leukopenia.<br><br>**Dengue with warning signs**<br>Patient who meets the above definition and also presents any of the following warning signs: Intense and continuous abdominal pain or tenderness, persistent vomiting, diarrhea, fluid accumulation (ascites, pleural effusion, pericardial effusion), mucosal bleeding, lethargy or irritability (mainly in children), postural hypotension, painful hepatomegaly >2 cm, drop in temperature, abrupt drop in platelets (<100,000) associated with hemoconcentration.<br><br>**Probable case of Severe Dengue**<br>Any case of dengue that meets any of the following serious manifestations of dengue: severe extravasation of plasma leading to dengue shock syndrome or fluid accumulation with respiratory distress; severe bleeding with hemodynamic compromise; clinical or paraclinical signs of severe organ damage such as liver damage, central nervous system damage, heart or other organ involvement.
**Confirmed case**	**By clinical epidemiological criteria**<br>Probable case residing within a perimeter of 200 meters (approximately two blocks) of another laboratory-confirmed case within 21 days (3 weeks) before or after laboratory diagnosis.<br><br>**By laboratory**<br>Probable case of dengue, severe dengue, or death due to dengue confirmed by any of the laboratory criteria for the diagnosis of dengue. PCR or viral isolation in patients with less than 5 days of onset of fever or IgM Dengue ELISA test in patients with 5 or more days of onset of fever (rapid tests are not accepted).
**CHIKUNGUNYA [2]**	
**Probable case**	Patient who resides or has visited 8 to 15 days before the onset of symptoms, a municipality located between 0 and 2,200 meters above sea level, where no cases of chikungunya have been confirmed by laboratory and who has a fever greater than 38°C, severe arthralgia or acute-onset arthritis, erythema multiforme, or symptoms not explained by other medical conditions.<br><br>Risk group patient who comes from areas located between 0 and 2,200 meters above sea level (regardless of whether or not they have confirmed viral circulation), 8 to 15 days before the onset of symptoms, who has a fever greater than 38°C, severe arthralgia or acute-onset arthritis and erythema multiforme or symptoms that

	are not explained by other medical conditions and from whom the sample is collected.
**Confirmed case**	**<u>By clinical epidemiological criteria</u>**<br>Patient with fever greater than 38°C, severe arthralgia or arthritis of acute onset, erythema multiforme or symptoms that are not explained by other medical conditions, who resides in or has visited a municipality where there is evidence of the circulation of the CHIKV virus, or is located in a municipality with a radius of 30 kilometers to municipalities with viral circulation.<br><br>**<u>By laboratory</u>**<br>Probable case with any of the following virus-specific laboratory tests with a positive result (viral isolation, RT-PCR, IgM), or a four-fold increase in the titer of specific IgG antibodies to chikungunya virus in paired samples with a difference of 15 days between taking these.
**ZIKA [3]**	
**Probable case**	Patient with rash and one or more of the following symptoms not explained by other medical conditions: fever not greater than 38.5°C, nonpurulent conjunctivitis or conjunctival hyperemia, arthralgia, myalgia, headache, or malaise. Additionally, one of the following conditions: i) Person who visited two weeks before the onset of symptoms countries or municipalities located between 0 and 2,200 m above sea level, with or without confirmed indigenous circulation of the Zika virus; ii) Person who had sexual contact without barrier protection two weeks before the onset of symptoms with a person who in the eight weeks prior to sexual contact visited areas with confirmed Zika transmission and/or areas with the presence of *Aedes* mosquitoes.
**Confirmed case**	**<u>By clinical epidemiological criteria</u>**<br>Patient who has been in countries or municipalities located between 0 and 2,200 meters above sea level with confirmed autochthonous circulation of the Zika virus two weeks before the onset of symptoms and who presents rash and one or more of the following symptoms that are not explained by other medical conditions: fever not higher than 38.5 °C, non-purulent conjunctivitis or conjunctival hyperemia, arthralgia, myalgia, headache or general malaise.<br><br>**<u>By laboratory</u>**<br>Case that meets the definition for probable case and that presents a positive result for Zika virus by RT-PCR (or immunohistochemistry in histopathological analysis) performed at the National Reference Laboratory of the National Institute of Health (INS) or collaborating centers designated by the INS.

References

1. Padilla JC, Rojas DP, Sáenz Gómez R. Dengue en Colombia: epidemiología de la reemergencia a la hiperendemia. 1st ed. Bogotá, D.C., Colombia; 2012.
2. Botero DS, Instituto Nacional de Salud, Colombia. Protocolo de vigilancia en salud pública – Chikungunya. 2017. Available: http://www.ssmcucuta.gov.co/observatorio/_lib/img/protocolos/pro_chikungunya.pdf.
3. Pérez NT. Protocolo de vigilancia en salud pública – Enfermedad por Virus Zika. 2017. Available: https://www.ins.gov.co/BibliotecaDigital/PRO-Zika.pdf
