# Supplementary material for: Spatio-temporal clusters and patterns of spread of dengue, chikungunya, and Zika in Colombia
Source: PLoS Negl Trop Dis. 2022 Aug 23;16(8):e0010334. doi: 10.1371/journal.pntd.0010334 (PMC9439233; doi:10.1371/journal.pntd.0010334)
Supplement: S2 Appendix — (PDF) [file pntd.0010334.s005.pdf]

Spatio-temporal clusters and patterns of spread of dengue, chikungunya, and Zika in Colombia

S2 Appendix

Laís P. Freitas, Mabel Carabali, Mengru Yuan, Gloria I. Jaramillo-Ramirez, Cesar G. Balaguera, Berta N. Restrepo, Kate Zinszer

Tested models for the front wave velocity analysis

The final models (one for each disease) are highlighted in red and were selected based on the Akaike Information Criterion (AIC), Bayesian information criterion (BIC) and expert judgment.

Table A. Zika spread velocity (average, 1st and 3rd quartiles) in kilometers per week estimated using trend surface analysis and polynomials order 1 to 9, and criteria for model selection, Colombia, 2014-2018.

	Velocity (km/week)			Model selection criteria	
Order	Average	Q1	Q3	AIC	BIC
1	21.53	21.12	21.94	5,898.74	5,921.96
2	222.95	70.28	224.76	5,883.60	5,916.11
3	138.68	57.81	171.88	5,880.11	5,921.90
4	84.01	45.64	90.19	5,877.82	5,928.90
**5**	**78.60**	**41.00**	**91.13**	**5,873.21**	**5,933.58**
6	70.48	38.24	77.06	5,875.11	5,944.77
7	53.61	25.90	58.14	5,866.53	5,945.48
8	51.22	25.60	51.74	5,856.78	5,945.01
9	39.87	21.32	41.43	5,848.85	5,946.37

Note:
Q1 = 1st quartile; Q3 = 3rd quartile; AIC = Akaike information criterion; BIC = Bayesian information criterion.

Table B. Chikungunya spread velocity (average, 1st and 3rd quartiles) in kilometers per week estimated using trend surface analysis and polynomials order 1 to 9, and criteria for model selection, Colombia, 2014-2018.

	Velocity (km/week)			Model selection criteria	
Order	Average	Q1	Q3	AIC	BIC
1	31.69	30.32	32.85	8,288.61	8,312.48
2	48.80	27.31	55.91	8,285.39	8,318.81
3	42.54	19.12	50.77	8,262.77	8,305.74
4	43.92	18.93	51.66	8,266.70	8,319.22
**5**	**27.18**	**11.73**	**31.18**	**8,216.39**	**8,278.46**
6	26.13	11.66	27.73	8,211.40	8,283.01
7	17.49	9.27	18.50	8,203.70	8,284.86
8	16.41	9.31	16.93	8,204.86	8,295.57
9	15.12	8.23	15.08	8,186.46	8,286.71

Note:

Q1 = 1st quartile; Q3 = 3rd quartile; AIC = Akaike information criterion; BIC = Bayesian information criterion.
