# Supplementary material for: Spatio-temporal clusters and patterns of spread of dengue, chikungunya, and Zika in Colombia
Source: PLoS Negl Trop Dis. 2022 Aug 23;16(8):e0010334. doi: 10.1371/journal.pntd.0010334 (PMC9439233; doi:10.1371/journal.pntd.0010334)
Supplement: S3 Appendix — (PDF) [file pntd.0010334.s006.pdf]

Spatio-temporal clusters and patterns of spread of dengue, chikungunya, and Zika in Colombia

S3 Appendix

Laís P. Freitas, Mabel Carabali, Mengru Yuan, Gloria I. Jaramillo-Ramirez, Cesar G. Balaguera, Berta N. Restrepo, Kate Zinszer

SaTScan parameters setting

This appendix describes the steps taken to define the values used for parameters setting of SaTScan (https://www.satscan.org/) [1-3]. Details of the methodology can be found in the main text.

1 Maximum population at risk inside a cluster

1.1 50% (standard)

The first analyses were explored using the standard settings for the cluster size (maximum population at risk of 50%) and a minimum of 2 cases inside the cluster (this is the minimum value allowed by SaTScan for this parameter).

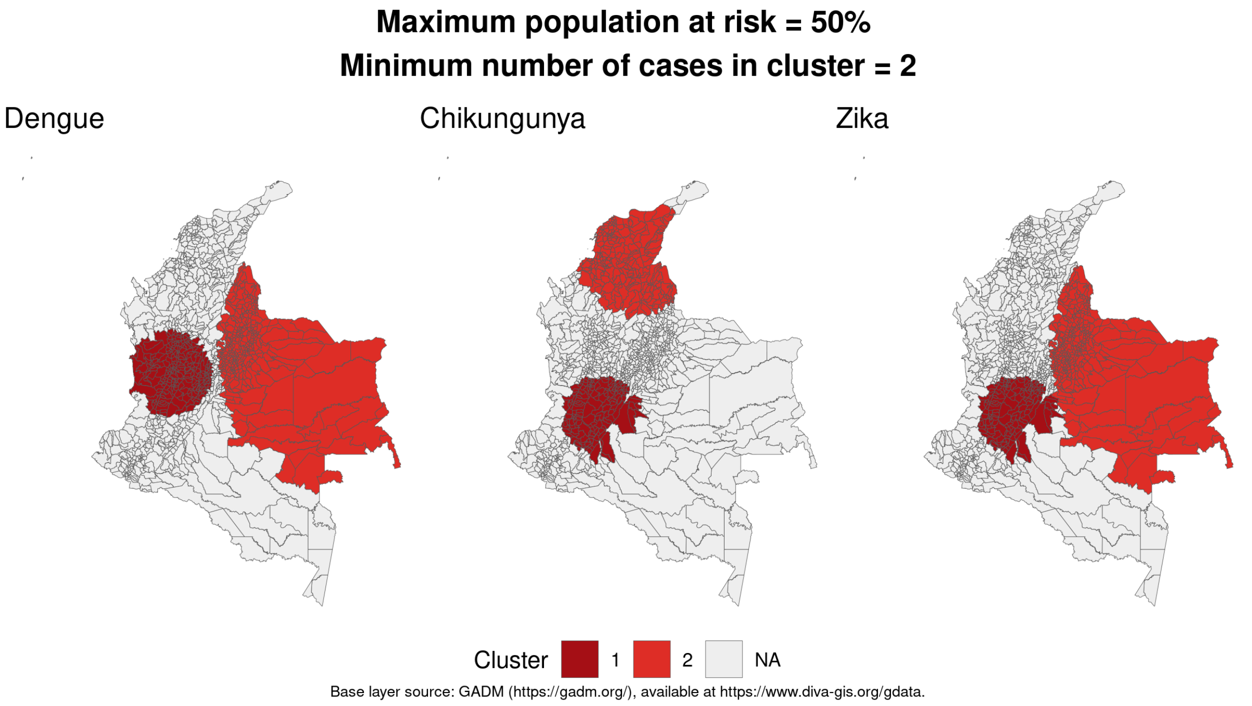

Two spatio-temporal clusters for each disease were detected. Next, we checked the relative risks of the municipalities that were part of a cluster, for the period the cluster was detected.

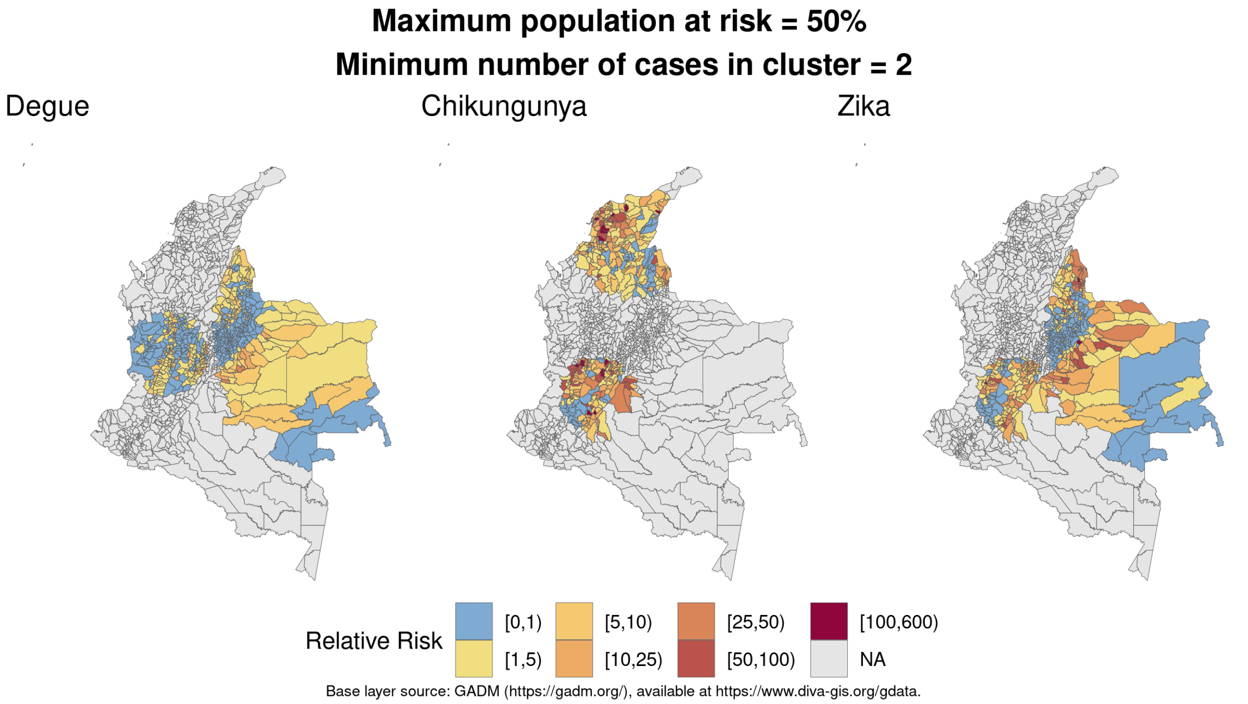

Of the 607 municipalities part of a dengue cluster, 333 (54.86%) had a relative risk < 1.0. Of the 389 municipalities part of a chikungunya cluster, 65 (16.71%) had a relative risk < 1.0. Of the 499 municipalities part of a Zika cluster, 235 (47.09%) had a relative risk < 1.0. We concluded that with the standard settings, too many municipalities with low relative risk were being considered as part of clusters for dengue and Zika. This happens because scan statistics uses circular scans to detect clusters, and, as a consequence, low-risk municipalities can be considered as part of a cluster if they are inside the scan area with high-risk municipalities.

1.2 Defining the maximum population at risk

To reduce the possibility of having low-risk municipalities in a cluster, we can limit the size of the reported clusters so that big clusters can be separated into smaller ones. It is also of interest to explore if sensibility increases and other high-risk areas can be detected.

Therefore, we wanted to define the maximum size of population at risk inside the cluster. To do that, we first needed to understand the population distribution across the municipalities of Colombia. As we can see in the plot below, the population distribution is highly skewed, with extreme values.

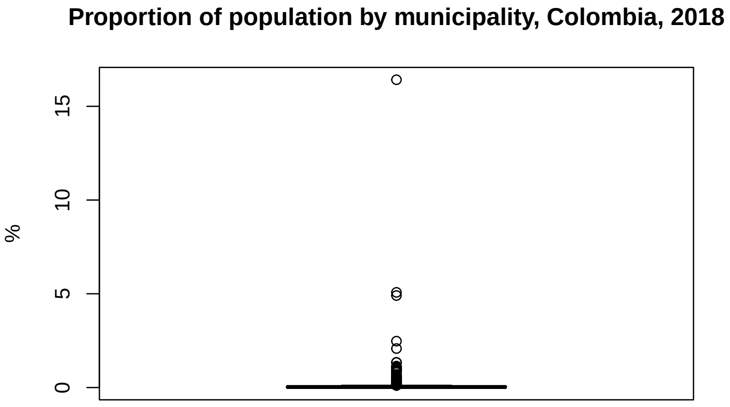

In 2018, the highest proportion of Colombia's population in one municipality was of 16.4%. Therefore, the maximum population at risk inside a cluster had to be higher than 16.4% so that no municipality of Colombia was excluded from the analyses. We rounded this value to 20% and reran SaTScan.

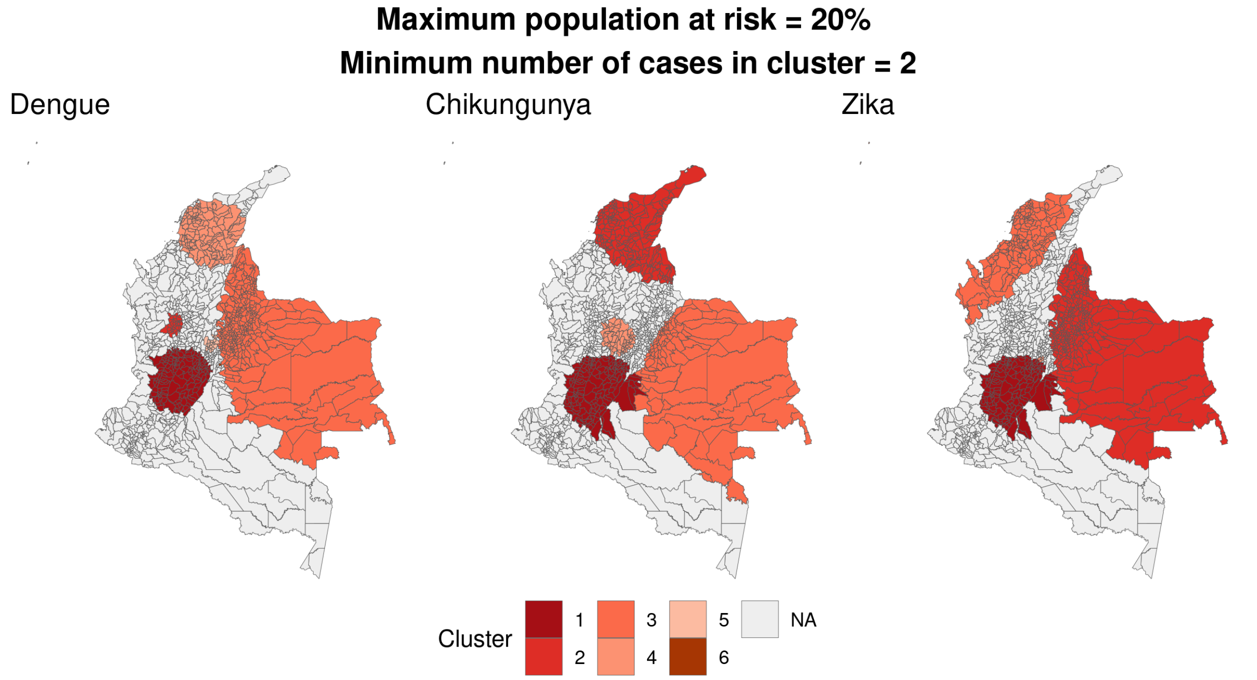

Base layer source: GADM (https://gadm.org/), available at https://www.diva-gis.org/gdata.

We identified 6 dengue clusters, 4 chikungunya clusters and 4 Zika clusters. We can observe that clusters of very different sizes were detected.

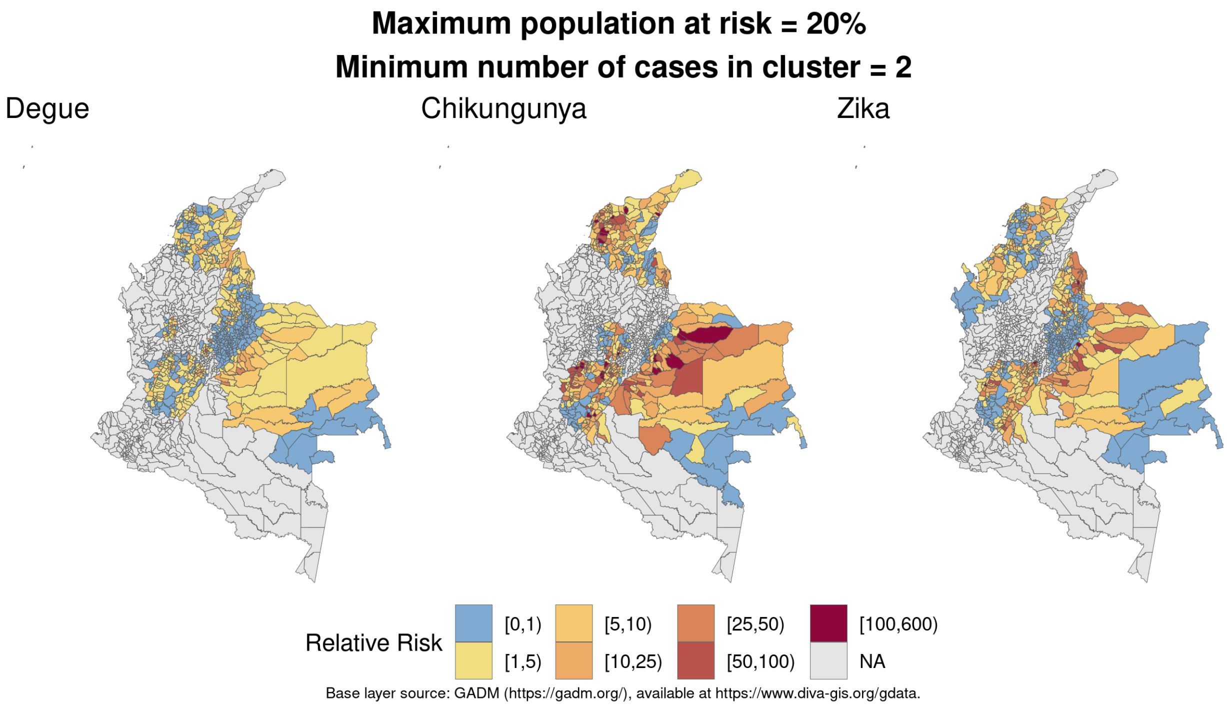

Of the 676 municipalities part of a dengue cluster, 328 (48.52%) had a relative risk < 1.0. For chikungunya, of the 523 municipalities part of a cluster, 135 (25.81%) had a relative risk < 1.0. And for Zika, of the 670 municipalities part of a dengue cluster, 299 (44.63%) had a relative risk < 1.0. Compared with the previous settings, we detected more clusters and more municipalities as part of high-risk clusters, while the clusters of dengue and Zika had smaller proportions of low-risk municipalities (relative risk < 1.0).

	**Maximum population inside cluster**	
	50%	20%
**Dengue**		
Total nº of municipalities in clusters	607	676
Nº of municipalities in clusters with RR<1.0	333	328
% of municipalities in clusters with RR<1.0	54.86	48.52
**Chikungunya**		
Total nº of municipalities in clusters	389	523
Nº of municipalities in clusters with RR<1.0	65	135
% of municipalities in clusters with RR<1.0	16.71	25.81
**Zika**		
Total nº of municipalities in clusters	499	670
Nº of municipalities in clusters with RR<1.0	235	299
% of municipalities in clusters with RR<1.0	47.09	44.63

However, the percentage of municipalities in clusters with relative risk < 1.0 is still high. Therefore, we decided to additionally restrict the size of the cluster using another parameter: the maximum radius of a cluster.

2 Maximum radius of a cluster

The following analyses were performed considering the maximum population inside the cluster of 20%, and minimum 2 cases in the cluster.

Without restricting the maximum size of the radius, the biggest cluster detected was for Zika and had a radius of 921.34 km (because it included in one cluster islands far off the Atlantic coast as well as municipalities within the mainland). After this one, big clusters were also detected in the west of Colombia, for the three diseases, each with radius of approximately 650 km. Therefore, to further restrict the size of the clusters and potentially decrease the percentage of municipalities in clusters with relative risk < 1.0, we started exploring different maximum radius sizes for the clusters, starting with 300 km.

2.1 300 km

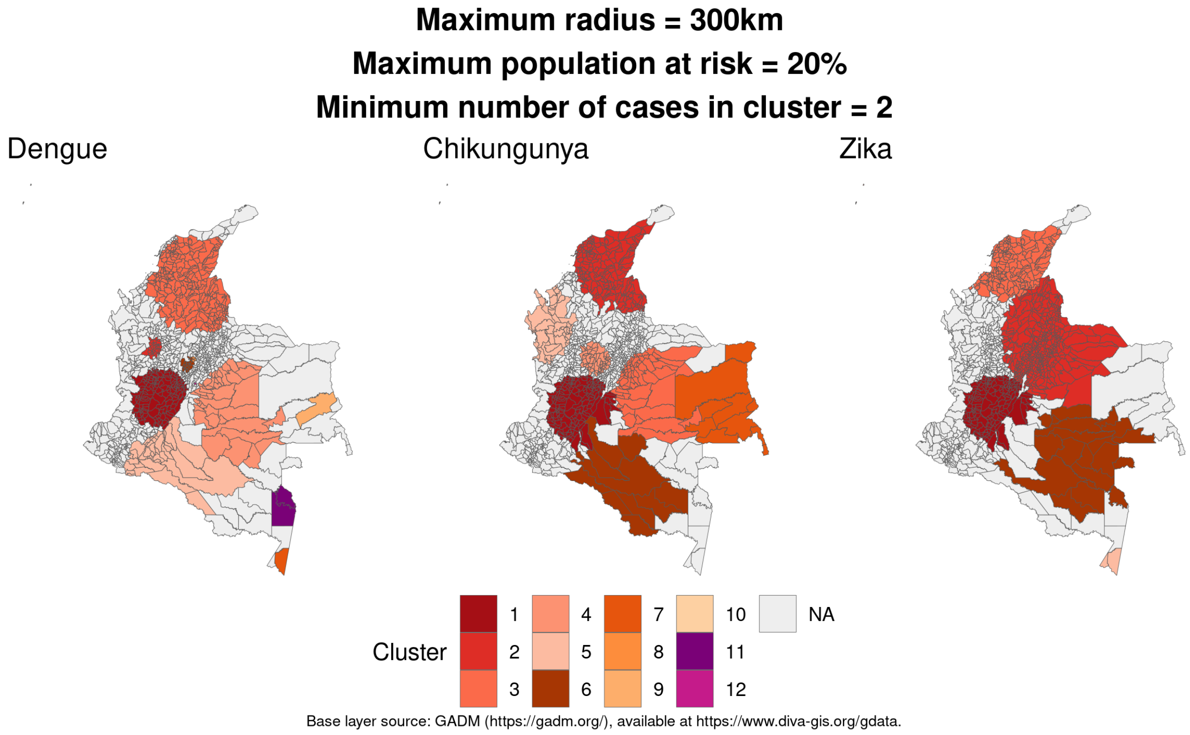

Under this setting, 12 dengue, 8 chikungunya and 6 Zika clusters were identified, of with less discrepant sizes than the previous setting. We can also observe that new regions of the country were identified as high-risk clusters.

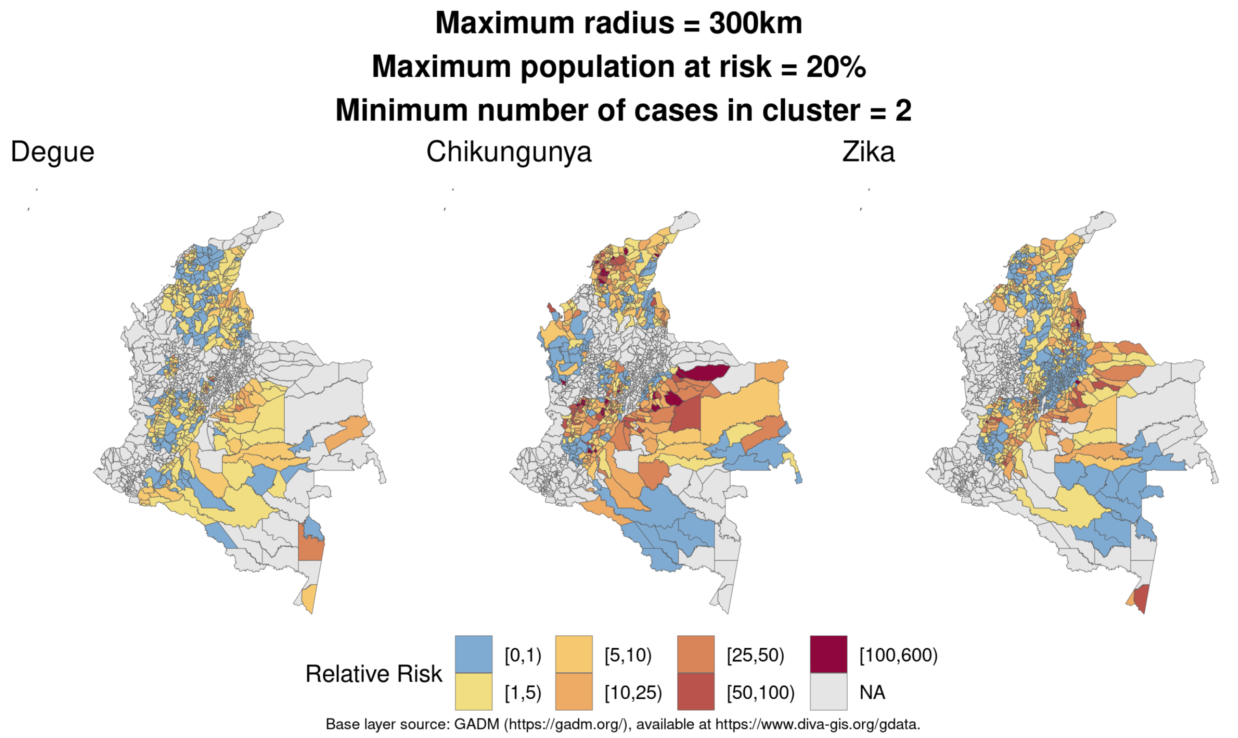

Of the 538 municipalities part of a dengue cluster, 172 (31.97%) had a relative risk < 1.0. For chikungunya, of the 550 municipalities part of a cluster, 146 (26.55%) had a relative risk < 1.0. And for Zika, of the 779 municipalities part of a dengue cluster, 299 (44.42%) had a relative risk < 1.0. Compared with the previous settings, we detected more clusters with smaller proportions of low-risk municipalities for dengue and Zika. For chikungunya, more clusters were detected, but the percentage of low-risk municipalities inside clusters had a small increase.

	**Maximum cluster radius**	
	unlimited	300 km
**Dengue**		
Nº of clusters	6	12
Total nº of municipalities in clusters	676	538
Nº of municipalities in clusters with RR<1.0	328	172
% of municipalities in clusters with RR<1.0	48.52	31.97
**Chikungunya**		
Nº of clusters	4	8
Total nº of municipalities in clusters	523	550
Nº of municipalities in clusters with RR<1.0	135	146
% of municipalities in clusters with RR<1.0	25.81	26.55
**Zika**		
Nº of clusters	4	6
Total nº of municipalities in clusters	670	779
Nº of municipalities in clusters with RR<1.0	299	346
% of municipalities in clusters with RR<1.0	44.63	44.42

2.2 150 km

Next, we explored restricting the maximum radius of a cluster at 150 km.

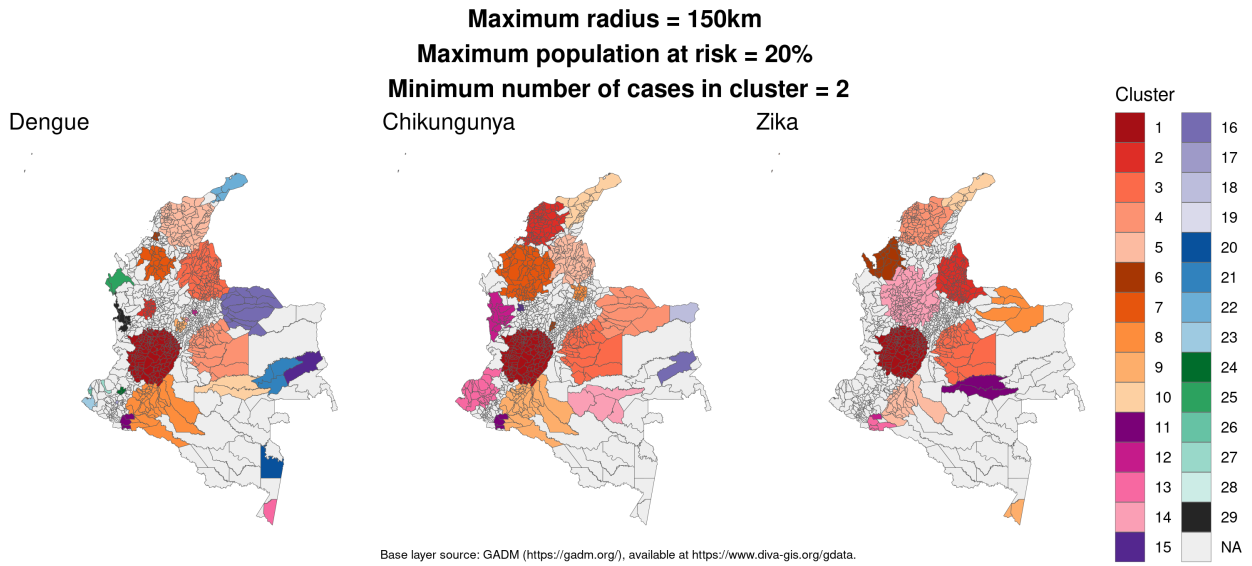

The number of identified clusters increased substantially, to 29 for dengue, 18 for chikungunya and 14 for Zika.

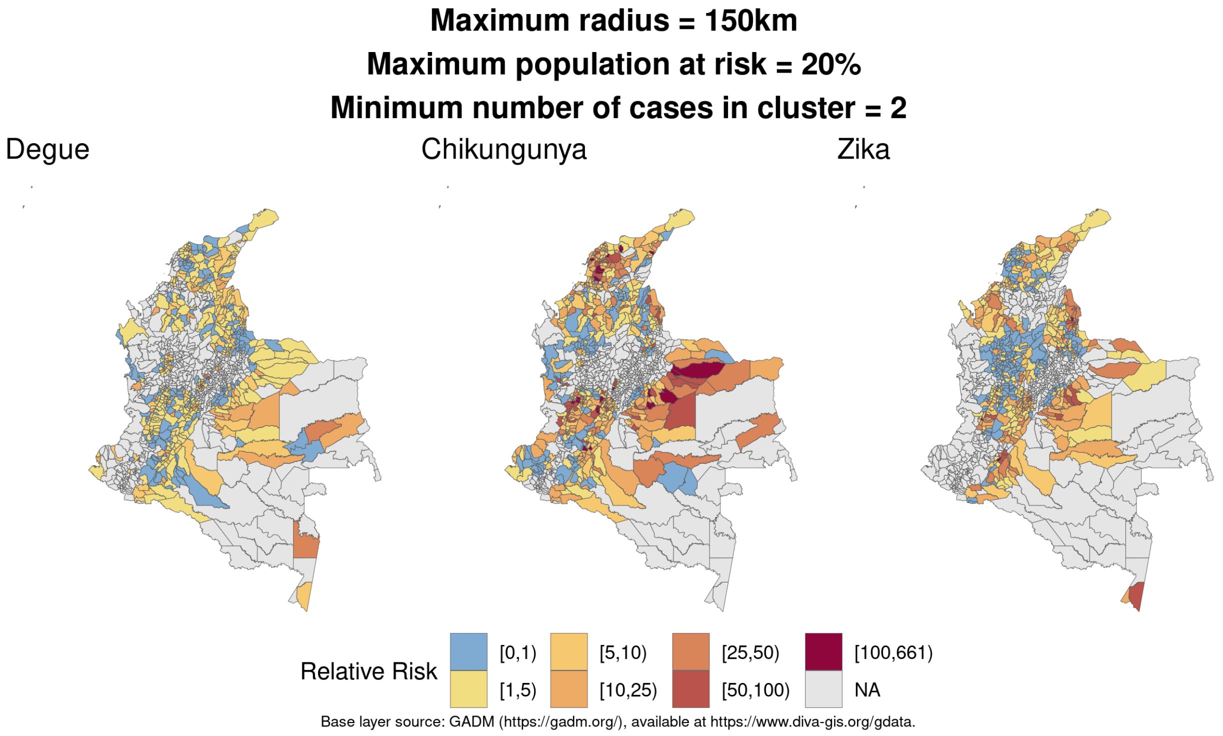

Of the 596 municipalities part of a dengue cluster, 200 (33.56%) had a relative risk < 1.0. For chikungunya, of the 629 municipalities part of a cluster, 167 (26.55%) had a relative risk < 1.0. And for Zika, of the 655

municipalities part of a dengue cluster, 257 (39.24%) had a relative risk < 1.0. Compared with the previous settings, smaller proportions of low-risk municipalities inside clusters were achieved only for Zika. For dengue and chikungunya, more clusters and more municipalities as part of clusters were identified, however there was a small decrease for the percentage of low-risk municipalities inside the cluster for dengue, while this percentage remained the same for chikungunya.

	**Maximum cluster radius**		
	unlimited	300 km	150 km
**Dengue**			
Nº of clusters	6	12	29
Total nº of municipalities in clusters	676	538	596
Nº of municipalities in clusters with RR<1.0	328	172	200
% of municipalities in clusters with RR<1.0	48.52	31.97	33.56
**Chikungunya**			
Nº of clusters	4	8	18
Total nº of municipalities in clusters	523	550	629
Nº of municipalities in clusters with RR<1.0	135	146	167
% of municipalities in clusters with RR<1.0	25.81	26.55	26.55
**Zika**			
Nº of clusters	4	6	14
Total nº of municipalities in clusters	670	779	655
Nº of municipalities in clusters with RR<1.0	299	346	257
% of municipalities in clusters with RR<1.0	44.63	44.42	39.24

2.3 100 km

Next, we explored restricting the maximum radius of a cluster at 100 km.

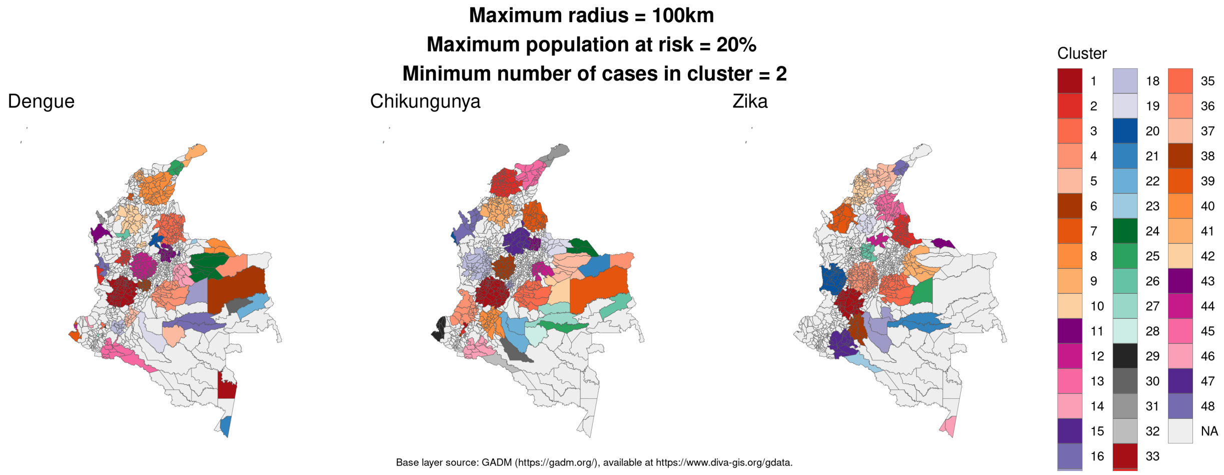

Note that in the figure above we had to repeat the colors after cluster #32.

The number of identified clusters almost doubled compared with using 150 km as the maximum radius of a cluster. We identified 48 dengue, 39 chikungunya and 26 Zika, clusters.

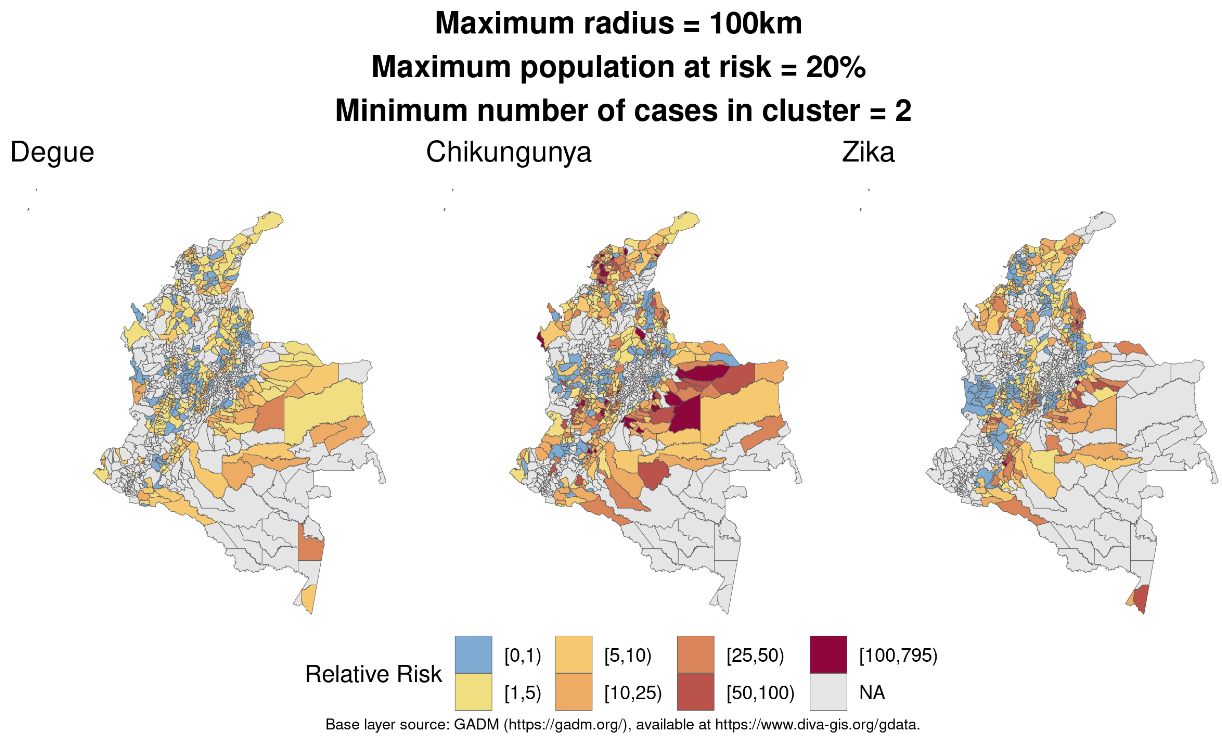

Despite the higher number of clusters detected, the number of municipalities inside clusters actually decreased compared with the previous setting (150 km). Compared with previous settings, the maximum radius of 100 km resulted in smaller percentage of low-risk municipalities inside clusters. However, we observed that with the maximum radius of 100 km neighboring high-risk municipalities were sometimes separated into different clusters, each one being constituted of a single municipality. This happened because the distance between the centroids of these neighboring municipalities exceeded the maximum cluster size in km.

2.4 Defining the maximum radius

For the reasons explained above, we considered that a maximum cluster radius of 100 km was too small for the municipalities of Colombia.

	Maximum cluster radius			
	unlimited	300 km	150 km	100 km
**Dengue**				
Nº of clusters	6	12	29	48
Total nº of municipalities in clusters	676	538	596	548
Nº of municipalities in clusters with RR<1.0	328	172	200	164
% of municipalities in clusters with RR<1.0	48.52	31.97	33.56	29.93
**Chikungunya**				
Nº of clusters	4	8	18	39
Total nº of municipalities in clusters	523	550	629	615
Nº of municipalities in clusters with RR<1.0	135	146	167	149
% of municipalities in clusters with RR<1.0	25.81	26.55	26.55	24.23
**Zika**				
Nº of clusters	4	6	14	26
Total nº of municipalities in clusters	670	779	655	571
Nº of municipalities in clusters with RR<1.0	299	346	257	200
% of municipalities in clusters with RR<1.0	44.63	44.42	39.24	35.03

Considering the results after experimenting the three different maximum radius sizes, we decided to continue with the maximum radius size of 150 km because, compared with the setting of 300 km, it detected more clusters while decreasing (for Zika) or maintaining (for chikungunya) the percentage of low-risk municipalities inside clusters. A small increase in the percentage of low-risk municipalities in dengue clusters were observed, but we concluded that this was outweighed by the other improvements.

3 Minimum cases inside a cluster

All analyses above were performed considering a minimum of 2 cases inside a cluster. Here, we explore the temporal distribution of cases inside the detected clusters using as parameters the maximum population at risk inside a cluster of 20% and the maximum radius size of 150 km:

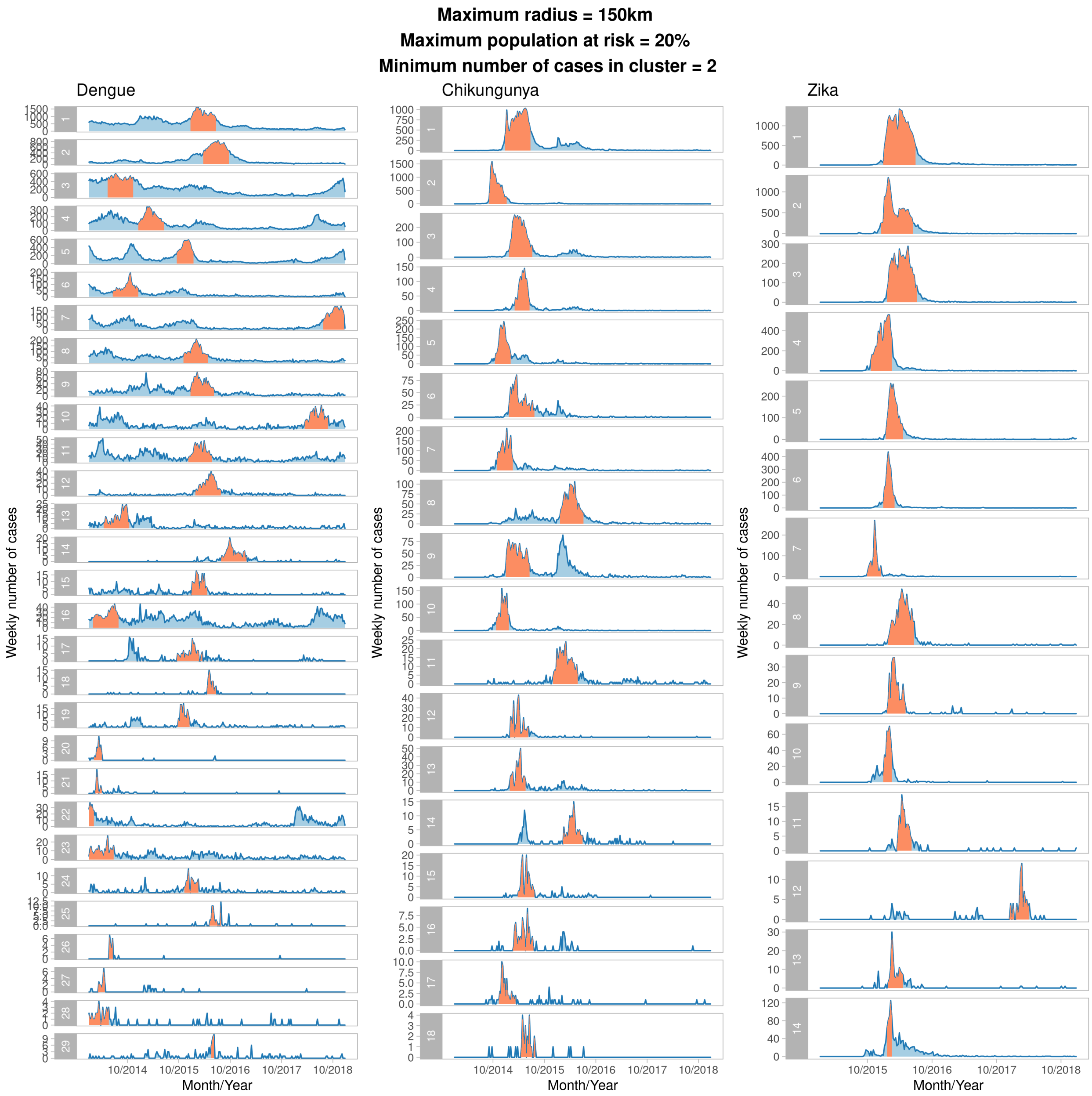

We observed that some clusters had sporadic cases and small total number cases, specially in the bottom of the likelihood ratio rank. Here is a summary of the number of cases inside clusters for each disease:

**Maximum radius = 150km, Minimum number of cases = 2**			
	**Nº of observed cases**		
	Mean	Min	Max
Dengue	3025.621	20	31569
Chikungunya	2797.611	23	20445
Zika	4329.643	68	26653

We performed the analyses also considering a minimum of 100 cases inside a cluster and compared with the analyses using a minimum of 2 cases.

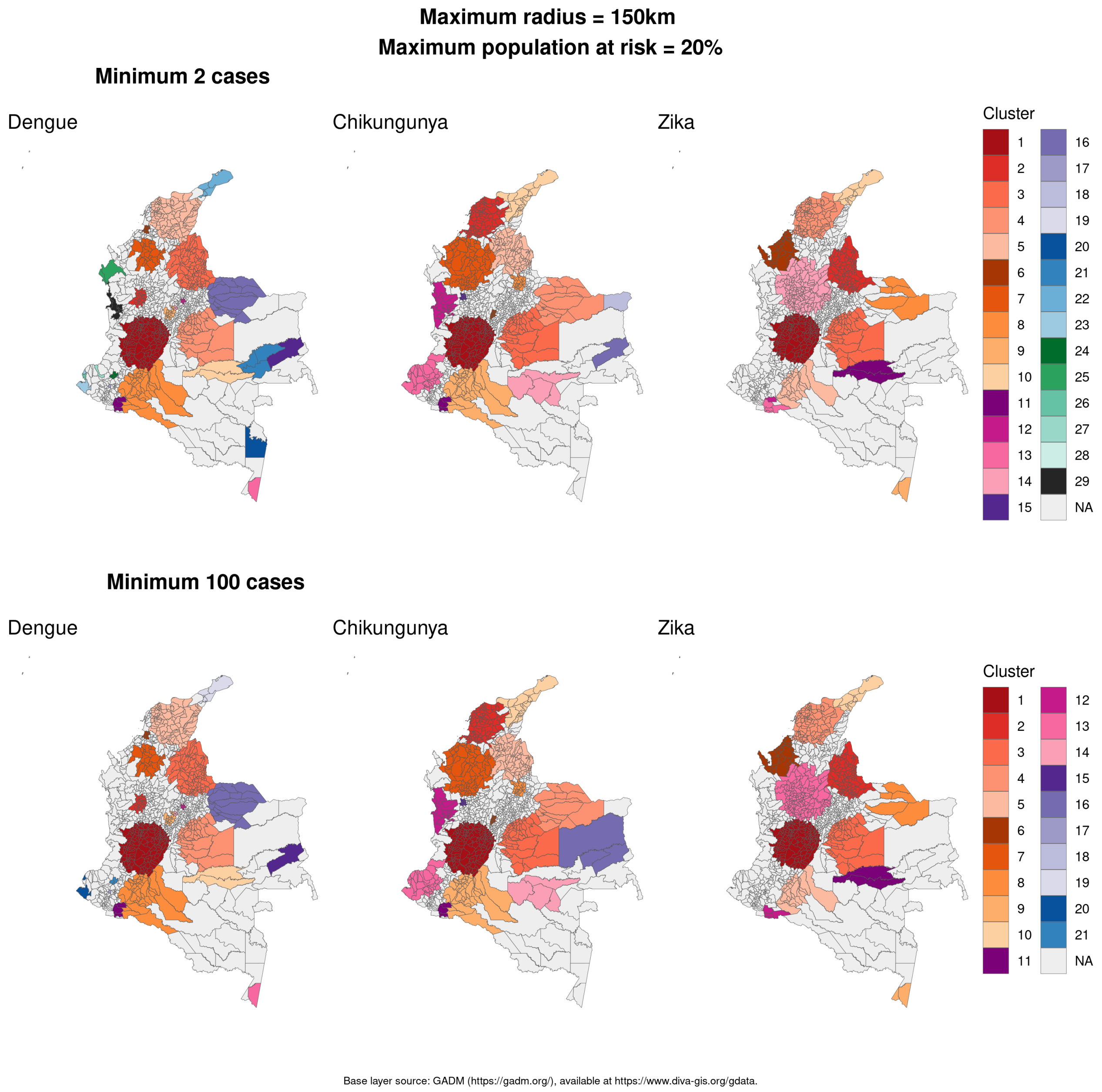

The maps are very similar, the majority of areas identified as clusters remained the same.

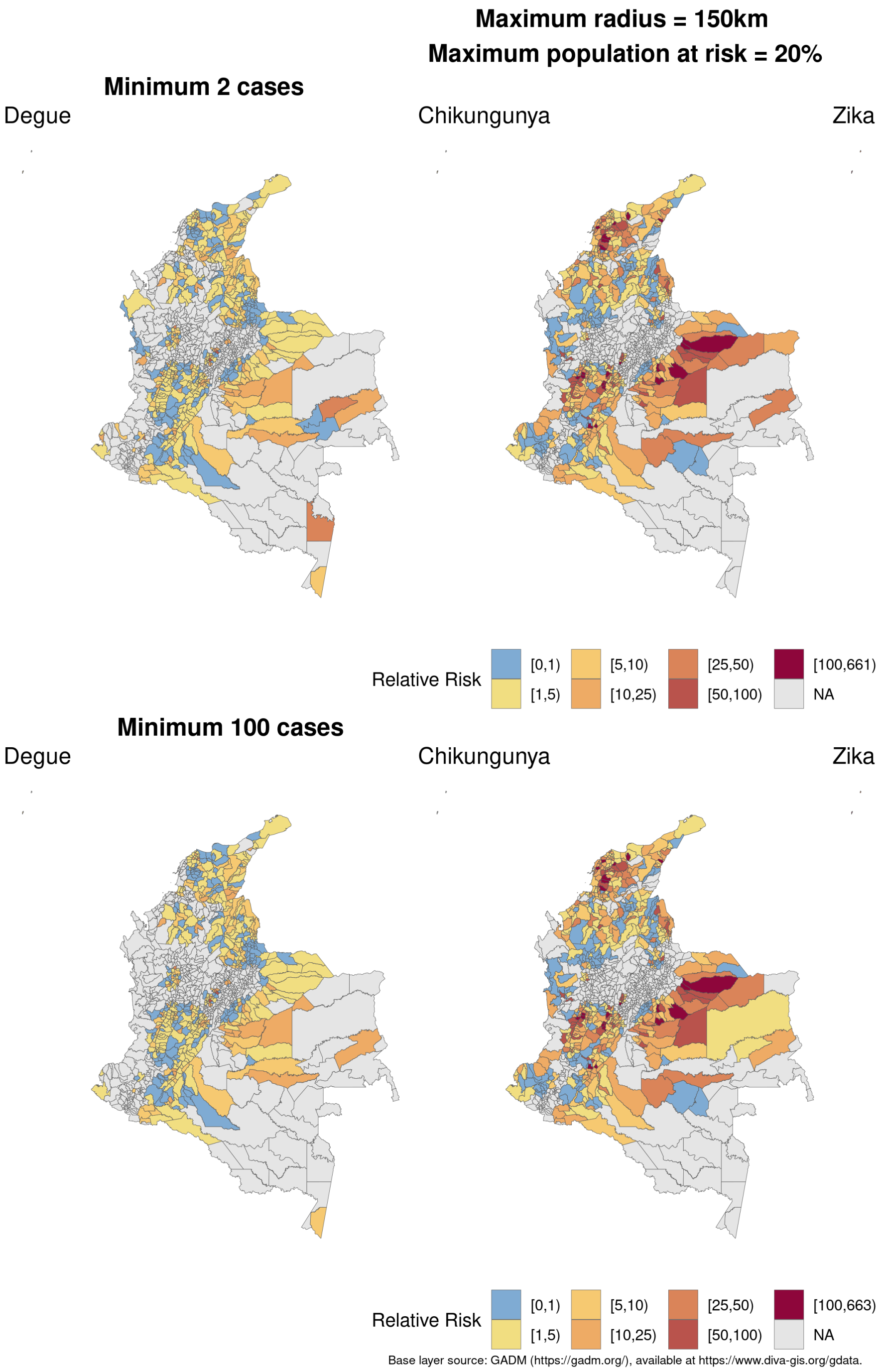

Of the 580 municipalities part of a dengue cluster, 193 (33.28%) had a relative risk < 1.0. For chikungunya, of the 629 municipalities part of a cluster, 167 (26.55%) had a relative risk < 1.0. And for Zika, of the 654 municipalities part of a dengue cluster, 257 (39.30%) had a relative risk < 1.0. The results are very similar to the ones using a minimum of 2 cases inside a cluster.

	Minimum nº of cases in cluster	
	2	100
**Dengue**		
Nº of clusters	29	21
Total nº of municipalities in clusters	596	580
Nº of municipalities in clusters with RR<1.0	200	193
% of municipalities in clusters with RR<1.0	33.56	33.28
**Chikungunya**		
Nº of clusters	18	16
Total nº of municipalities in clusters	629	629
Nº of municipalities in clusters with RR<1.0	167	167
% of municipalities in clusters with RR<1.0	26.55	26.55
**Zika**		
Nº of clusters	14	13
Total nº of municipalities in clusters	655	654
Nº of municipalities in clusters with RR<1.0	257	257
% of municipalities in clusters with RR<1.0	39.24	39.3

Finally, the summary of the number of cases inside clusters for each disease and the weekly number of cases inside each cluster considering a minimum of 100 cases in a cluster:

Maximum radius = 150km, Minimum number of cases = 100			
	Nº of observed cases		
	Mean	Min	Max
Dengue	4164.476	100	31569
Chikungunya	3144.375	100	20445
Zika	4657.462	115	26653

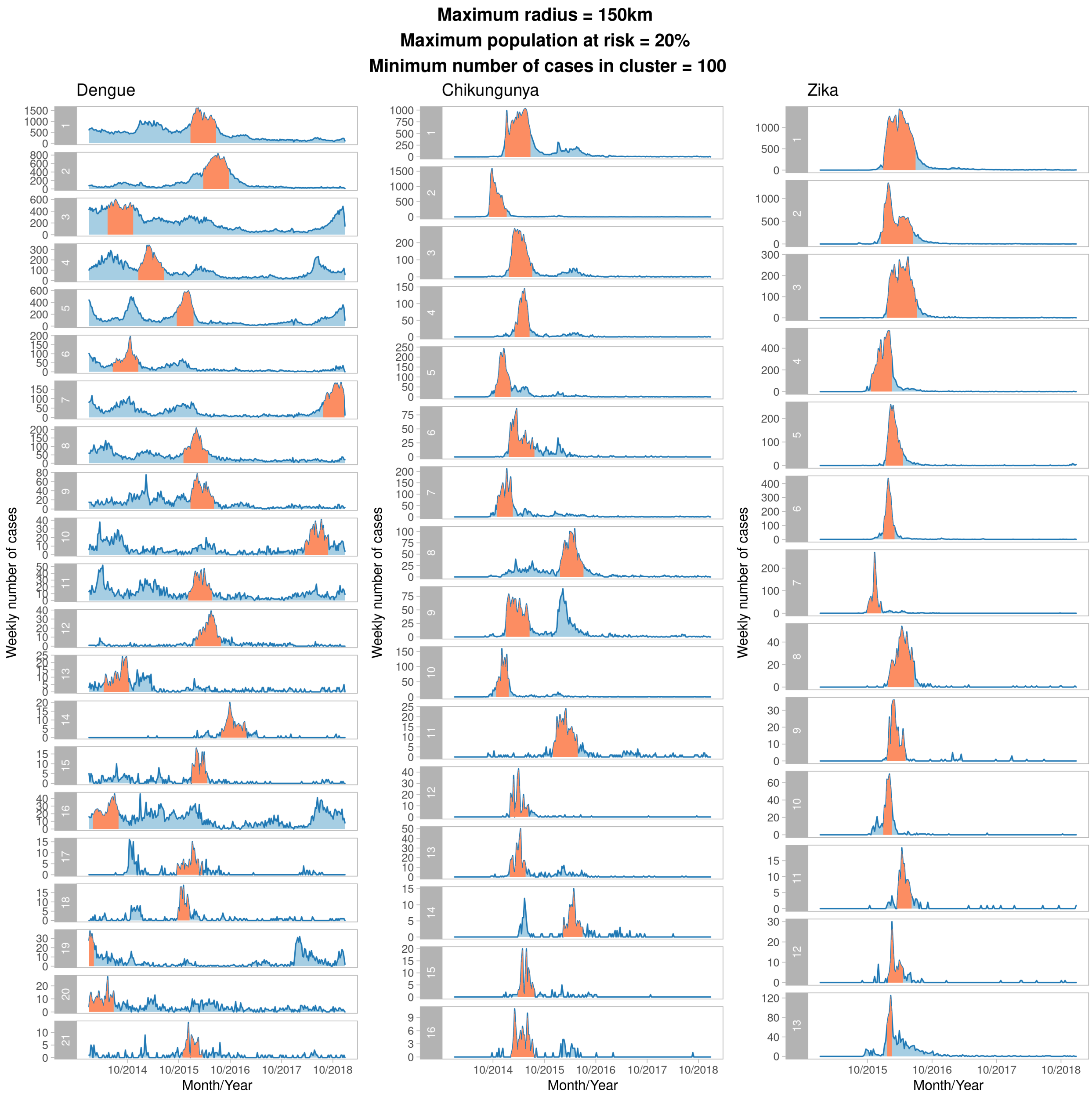

As a conclusion, modifying the minimum number of cases inside a cluster from 2 to 100 did not impact substantially the results of the scan statistics analyses, considering a maximum radius of 150km and a maximum population at risk of 20%.

References

1. Kulldorff M. SaTScan. Available: https://www.satscan.org/

2. Kulldorff M. 2021. SaTScan™ user guide for version 10.0. Available: https://www.satscan.org/cgi-bin/satscan/register.pl/SaTScan_Users_Guide.pdf?todo=process_userguide_download

3. Kulldorff M. A spatial scan statistic. Commun Stat - Theory Methods. 1997;26: 1481–1496. doi: 10.1080/03610929708831995
