# Supplementary material for: Spatio-temporal clusters and patterns of spread of dengue, chikungunya, and Zika in Colombia
Source: PLoS Negl Trop Dis. 2022 Aug 23;16(8):e0010334. doi: 10.1371/journal.pntd.0010334 (PMC9439233; doi:10.1371/journal.pntd.0010334)
Supplement: S1 Table — (PDF) [file pntd.0010334.s007.pdf]

Spatio-temporal clusters and patterns of spread of dengue, chikungunya, and Zika in Colombia

Laís P. Freitas, Mabel Carabali, Mengru Yuan, Gloria I. Jaramillo-Ramirez, Cesar G. Balaguera, Berta N. Restrepo, Kate Zinszer

S1 Table. Space-time clusters of dengue cases, Colombia, 2014-2018.

Cluster*	Time period (EWs)	Duration (weeks)	Population	Observed cases	Relative risk
1	51/2015 to 25/2016	27	8,514,425	31,569	5.30
2	12/2016 to 38/2016	27	4,034,942	16,933	5.80
3	20/2014 to 46/2014	27	3,532,385	12,815	5.04
4	51/2014 to 24/2015	27	1,234,977	5,534	6.15
5	37/2015 to 2/2016	18	5,257,948	7,561	3.00
6	25/2014 to 51/2014	27	369,699	2,188	8.13
7	30/2018 to 51/2018	22	1,352,215	2,976	3.53
8	44/2015 to 17/2016	26	1,213,266	2,903	3.34
9	51/2015 to 23/2016	25	188,231	1,067	8.19
10	11/2018 to 35/2018	25	88,976	575	9.03
11	49/2015 to 21/2016	25	127,491	639	7.25
12	4/2016 to 30/2016	27	68,778	508	9.84
13	16/2014 to 42/2014	27	41,638	294	9.55
14	30/2016 to 4/2017	27	16,819	204	16.16
15	52/2015 to 16/2016	17	19,979	162	17.54
16	5/2014 to 31/2014	27	365,074	699	2.60
17	37/2015 to 11/2016	27	20,899	136	8.75
18	38/2015 to 50/2015	13	71,943	129	5.22
19	1/2014 to 6/2014	6	500,857	181	2.58
20	1/2014 to 26/2014	26	203,979	281	1.98
21	43/2015 to 11/2016	21	62,340	100	2.79

* Ranked by likelihood ratio, being the first cluster the one with the maximum likelihood ratio.
EW = Epidemiological weeks
