# Supplementary material for: Spatio-temporal clusters and patterns of spread of dengue, chikungunya, and Zika in Colombia
Source: PLoS Negl Trop Dis. 2022 Aug 23;16(8):e0010334. doi: 10.1371/journal.pntd.0010334 (PMC9439233; doi:10.1371/journal.pntd.0010334)
Supplement: S2 Table — (PDF) [file pntd.0010334.s008.pdf]

Spatio-temporal clusters and patterns of spread of dengue, chikungunya, and Zika in Colombia

Laís P. Freitas, Mabel Carabali, Mengru Yuan, Gloria I. Jaramillo-Ramirez, Cesar G. Balaguera, Berta N. Restrepo, Kate Zinszer

S2 Table. Space-time clusters of chikungunya cases, Colombia, 2014-2018.

Cluster*	Time period (EWs)	Duration (weeks)	Population	Observed cases	Relative risk
1	52/2014 to 25/2015	27	8,514,425	20,445	20.39
2	36/2014 to 1/2015	19	5,707,750	13,850	27.27
3	3/2015 to 27/2015	25	1,308,450	4,440	24.94
4	9/2015 to 24/2015	16	298,693	1,300	48.90
5	42/2014 to 5/2015	17	2,032,985	2,207	11.59
6	3/2015 to 29/2015	27	84,771	984	74.48
7	44/2014 to 7/2015	17	3,046,326	1,881	6.64
8	3/2016 to 27/2016	25	1,324,008	1,469	7.71
9	53/2014 to 24/2015	25	1,213,266	1,313	7.61
10	43/2014 to 3/2015	14	2,013,751	1,115	7.37
11	48/2015 to 21/2016	26	127,491	341	17.67
12	4/2015 to 23/2015	20	311,920	290	8.14
13	4/2015 to 20/2015	17	736,860	333	4.72
14	6/2016 to 26/2016	21	50,348	116	18.92
15	12/2015 to 29/2015	18	87,547	126	13.88
16	5/2015 to 31/2015	27	62,632	100	10.26

* Ranked by likelihood ratio, being the first cluster the one with the maximum likelihood ratio.
EW = Epidemiological weeks
