# Supplementary material for: Spatio-temporal clusters and patterns of spread of dengue, chikungunya, and Zika in Colombia
Source: PLoS Negl Trop Dis. 2022 Aug 23;16(8):e0010334. doi: 10.1371/journal.pntd.0010334 (PMC9439233; doi:10.1371/journal.pntd.0010334)
Supplement: S3 Table — (PDF) [file pntd.0010334.s009.pdf]

Spatio-temporal clusters and patterns of spread of dengue, chikungunya, and Zika in Colombia

Laís P. Freitas, Mabel Carabali, Mengru Yuan, Gloria I. Jaramillo-Ramirez, Cesar G. Balaguera, Berta N. Restrepo, Kate Zinszer

S3 Table. Space-time clusters of Zika cases, Colombia, 2015-2018.

Cluster*	Time period (EWs)	Duration (weeks)	Population	Observed cases	Relative risk
1	52/2015 to 26/2016	27	8,602,402	26,653	24.67
2	50/2015 to 24/2016	27	3,375,936	16,005	31.34
3	3/2016 to 27/2016	25	1,319,458	4,663	21.43
4	42/2015 to 7/2016	18	6,833,462	6,181	7.89
5	2/2016 to 16/2016	15	857,738	2,096	24.53
6	52/2015 to 9/2016	10	1,869,732	2,130	17.84
7	39/2015 to 50/2015	12	77,429	933	152.53
8	4/2016 to 25/2016	22	140,082	658	30.81
9	3/2016 to 18/2016	16	50,140	263	47.85
10	52/2015 to 7/2016	8	940,273	348	7.32
11	11/2016 to 23/2016	13	89,715	115	14.64
12	4/2016 to 16/2016	13	195,768	122	7.12
13	3/2016 to 7/2016	5	6,607,483	380	1.97

* Ranked by likelihood ratio, being the first cluster the one with the maximum likelihood ratio.
EW = Epidemiological weeks
