# Supplementary material for: Spatio-temporal clusters and patterns of spread of dengue, chikungunya, and Zika in Colombia
Source: PLoS Negl Trop Dis. 2022 Aug 23;16(8):e0010334. doi: 10.1371/journal.pntd.0010334 (PMC9439233; doi:10.1371/journal.pntd.0010334)
Supplement: S4 Table — (PDF) [file pntd.0010334.s010.pdf]

Spatio-temporal clusters and patterns of spread of dengue, chikungunya, and Zika in Colombia

Laís P. Freitas, Mabel Carabali, Mengru Yuan, Gloria I. Jaramillo-Ramirez, Cesar G. Balaguera, Berta N. Restrepo, Kate Zinszer

S4 Table. Space-time clusters of dengue, chikungunya and Zika cases detected using multivariate scan statistics, Colombia, 2014-2018.

Cluster*	Time period (EWs)	Duration (weeks)	Population	Dengue RR	Chikungunya RR	Zika RR
1	52/2015 to 26/2016	27	8,580,330	5.19	3.19	30.92
2	49/2015 to 23/2016	27	3,364,640	2.73	3.33	39.21
3	36/2014 to 51/2014	16	5,516,025	2.61	31.41	NA
4	12/2016 to 38/2016	27	4,034,942	5.80	NA	NA
5	53/2014 to 26/2015	27	1,308,450	5.88	23.27	NA
6	52/2015 to 17/2016	18	1,213,266	3.65	6.69	20.17
7	52/2015 to 9/2016	10	1,852,593	1.03	NA	22.28
8	39/2015 to 50/2015	12	71,943	5.38	1.88	201.32
9	6/2015 to 24/2015	19	298,693	2.64	42.60	NA
10	1/2015 to 27/2015	27	84,771	7.13	73.95	NA
11	44/2015 to 7/2016	16	2,013,751	1.79	NA	9.72
12	48/2015 to 21/2016	26	127,491	7.06	17.67	3.68
13	10/2015 to 23/2015	14	2,166,569	NA	6.24	NA
14	3/2016 to 18/2016	16	49,917	1.85	3.28	59.76
15	11/2018 to 35/2018	25	88,976	9.03	NA	NA
16	4/2015 to 23/2015	20	311,920	NA	8.14	NA
17	52/2015 to 16/2016	17	19,979	17.54	10.77	1.06
18	6/2014 to 14/2014	9	5,127	34.80	NA	NA
19	7/2014 to 14/2014	8	8,968	22.94	NA	NA
20	15/2015 to 30/2015	16	16,000	2.93	15.96	NA

* Ranked by likelihood ratio, being the first cluster the one with the maximum likelihood ratio.
EW = Epidemiological weeks
RR = Relative risk
