# Supplementary material for: Spatio-temporal clusters and patterns of spread of dengue, chikungunya, and Zika in Colombia
Source: PLoS Negl Trop Dis. 2022 Aug 23;16(8):e0010334. doi: 10.1371/journal.pntd.0010334 (PMC9439233; doi:10.1371/journal.pntd.0010334)
Supplement: Attachment — Submitted filename: response_comments PNTDs.pdf [file pntd.0010334.s011.pdf]

Dr. Alberto Novaes Ramos Jr
Associate Editor
PLOS Neglected Tropical Diseases

Dr. Victor S. Santos
Deputy Editor
PLOS Neglected Tropical Diseases

17 June 2022

Dear Dr. Ramos and Dr. Santos,
Thank you for considering for publication our manuscript entitled "*Spatio-temporal clusters and patterns of spread of dengue, chikungunya, and Zika in Colombia*". I am pleased to submit a new version of the manuscript after consideration of the reviewers' comments.

We would also like to thank the reviewers for their time spent on reviewing our manuscript and their comments helping us improving the manuscript.

Please find below our responses to reviewer comments including a description of the changes made in the manuscript.

Sincerely,
Laís Picinini Freitas, PhD
École de Santé Publique - Université de Montréal
Centre de recherche en santé publique

Spatio-temporal clusters and patterns of spread of dengue, chikungunya, and Zika in Colombia

Plos NTDs
Responses to the review comments

Reviewer #1:

> Freitas et al. describe the epidemiology of the triple epidemic of Zika, chikungunya, and dengue in Colombia. To do this they identify at-risk regions using spatiotemporal scan statistics, and estimate the speed of spread using trend surface analysis. The methods are clearly described and are appropriate to address the study's objectives. The study population is clearly described (although I was a little unclear on the definition of a case - see below), and I have no concerns about ethical requirements as the study used publicly available data. The specific concerns relating to the methodology that I would like to be addressed are:
> - L107-113: I find the definition of a case a little confusing, please could you re-write this section. In particular it is unclear what category a positive antibody test would be in, as it is included in both definitions.
> - Related to this, please could you describe how you used probable vs confirmed cases and what the breakdown of these were (i.e. the proportion in each group).

We rewrote the paragraph with the case definition and have included a supporting material with the complete definitions for probable and confirmed cases for each disease. We believe that it is now clear that the data include both probable and confirmed cases, and it is not possible to distinguish between them because the data is aggregated. Therefore, we did not use probable vs confirmed cases.

Lines 123-128 (updated version):

“The aggregate data include probable and confirmed cases. Probable cases are those that present clinical signs within the case definition of each disease, following the official protocols of the National Institute of Health of Colombia [31–33]. In Colombia, probable cases can be confirmed either by laboratory (based upon a positive result from antigen, antibody, or virus detection and/or isolation) or by clinical and epidemiological criteria. Complete case definitions for probable and confirmed cases for each disease are available in the S1 Appendix.”

> - L128+: For the trend surface analysis, it would be useful to see the other models tested, perhaps as a supplementary table. In this table it would be useful to include the model tested, the BIC of each model, and the average and range of the estimated velocities. Including this would help the reader interpret these results by being able to compare across models.

Thank you for this comment. We have included a supplementary appendix (S2 Appendix)

with the tested models and their velocity estimates (average, 1st and 3rd quartiles), as well as their BIC and AIC.

- Please could you include a GitHub link to the code used to run the analyses.

The codes and files used to run the analysis are now available at https://github.com/laispfreitas/Colombia_DZC_satscan_velocity.

We also included this information in the text (updated version, lines 200-202):

> "The R codes and the data used in the analyses are available at https://github.com/laispfreitas/Colombia_DZC_satscan [47]."

The analysis matches the analysis plan and the results are clearly and sufficiently presented. I only have two comments:
- Could you label individually the importation locations which you mentioned in the text, so that a reader can find them.

We are assuming this comment refers to Fig 2. We include the labels in Fig 2, as suggested.

- Figs 3 and 4 captions, I assume you rank all clusters by their likelihood (not just the first)? Perhaps better to write clusters are ranked by likelihood than the first cluster is most likely if so. Or if not, it would be better to rank them this way I think.

In both captions it is stated that the clusters are ranked by likelihood ratio. The observation of the first cluster being the most likely cluster is to provide the information of the direction of the rank. We added the information "with the maximum likelihood ratio":

> "Fig 3. Space-time clusters ranked by likelihood ratio* (A-C), year of start date (D-F), duration in weeks (G-H) and relative risk (J-L) for dengue (1st column), chikungunya (2nd column) and Zika (3rd column), Colombia, 2014-2018. * The first cluster is the most likely cluster, i.e., with the maximum likelihood ratio."

> "Fig 5. Multivariate space-time clusters of dengue, chikungunya and Zika ranked by likelihood ratio* (A), diseases (B), year of start date (C), duration in weeks (D) and relative risk for each disease (E-G), Colombia, 2014-2018. * The first cluster is the most likely cluster, i.e., with the maximum likelihood ratio."

Note: We have included a new figure in the main text, so in the updated version the Fig 5 is the Fig 4 from the previous version.

The conclusions are generally supported by the results, and the limitations are well described. I have a few comments:
- In the limitations you touch upon the effect of isolated cases, but I wonder if you could also discuss the effect of repeated introductions and how this might affect your trend surface analysis in particular.

If there are different points of introduction, this would be seen in the model as it is based on temporal order of occurrence. For example, if different municipalities reported a first case of Zika in the same week, depending on the granularity of the temporal scale displayed on the map (e.g., 1-3 week units), this would be visible. We can see this on Fig 2A, and repeated introductions are likely the cause of the underlying patterns.

- L268: as your model is not predictive but descriptive, please say "was likely the place of emergence" here, rather than "is likely the place of emergence".
- L279: could you briefly describe what this other study did and what they found (one sentence)
- L280: "quicker" -> "shorter"
- L266: Please remove the sentence were you say "To our knowledge, this is the first study..." as it is not informative and hard to verify.

Thank you. We made modifications in the text as per the reviewer's suggestions.

Line 279 (previous version): We included more information for this study (lines 311-314 updated version):

> "Zika's accelerated spread is consistent with findings from another study using gravity models. The authors estimated that the spread of Zika was twice as fast as that of chikungunya, taking on average 15.7 weeks to invade half of the affected cities compared to 34.4 weeks for chikungunya [53]."

Other comment:
Abstract:
Please rewrite the third (Conclusions) section of the abstract. In particular, 1. remove the statement "To our knowledge, this is the first study...", and 2. instead of stating that this study furthers our knowledge, explain briefly how it furthers our knowledge / what knowledge it adds.

We rewrote the "Conclusions/Significance" section of the abstract (lines 33-36 updated version):

> "The results further our understanding of emerging *Aedes*-borne diseases in Colombia by providing useful evidence on their potential site of entry and spread trajectory within the country, and identifying spatio-temporal disease-specific and multivariate high-risk clusters of dengue, chikungunya, and Zika, information that can be used to target interventions."

Reviewer #2:

In SatScan the authors performed a retrospective analysis of spatiotemporal clusters in Colombia. It would be interesting to see the spatial variations in the temporal trend clusters. The spatial variation in the temporal trends identifies the clusters with growth rates (or decreases) in and out of these clusters, and evaluates whether the difference between them is statistically significant.

Thank you for this comment. The proposed analysis (spatial variations in the temporal trend clusters) would indeed be interesting although this would require identifying clusters with distinct temporal trends, compared to the rest of the study area. This is beyond the scope of our objectives for this study, as we are interested in identifying high-risk areas and not distinct temporal trends. However, it is something that we will be exploring in future work.

The results and figures are clearly and completely presented.

Thank you.

The conclusions are supported by the data presented.

Thank you.

line 55: which species of Aedes is incriminated to transmit these arboviruses in Colombia?

Now it is stated that it is the *Aedes aegypti* mosquito.

Manuscript needs a full proofread. Text writing needs to be more fluid and easier-to-read. Maybe join similar sentences in a more organic passage without repeating the same words so much, and also create acronyms for dengue, Zika and chikungunya.

Thank you. Full proofreading in the manuscript was done.

Can you clarify better the methodology applied to collect data? Were only confirmed cases considered? Or all those reported, even as suspects?

We used surveillance data, as explained in lines 100-102 (updated version):

> "This is an ecological study in which notified cases of dengue, chikungunya, and Zika from January 2014 to December 2018 for the entire country were extracted from the national surveillance system."

We rewrote the paragraph with the case definition. We believe that it is now clear that the data include probable and confirmed cases. Complete case definitions are now included in a supporting material (S1 Appendix).
Lines 117-128 (updated version):

> "In Colombia, surveillance is the responsibility of each department's Secretariat of Health although there is a national surveillance program administered by the National Institute of Health of Colombia that receives weekly reports from all health facilities that provide services to probable and confirmed cases of dengue, chikungunya, and Zika. The electronic platform, SIVIGILA (Sistema Nacional de Vigilancia en Salud Pública, http://portalsivigila.ins.gov.co/), provides publicly available aggregate data on chikungunya, dengue, and Zika cases at the municipal and departmental levels [35]. The aggregate data include probable and confirmed cases. Probable cases are those that present clinical signs within the case definition of each disease, following the official protocols of the National Institute of Health of Colombia [31–33]. In Colombia, probable cases can be confirmed either by laboratory (based upon a positive result from antigen, antibody, or virus detection and/or isolation) or by clinical and epidemiological criteria. Complete case definitions for probable and confirmed cases for each disease are available in the S1 Appendix."

Regarding Kulldorff's scan statistics, the authors used GINI index for optimization?

The Gini index can be used to define the cluster reporting sizes in SaTScan. However, according to SaTScan User Guide (version 10.0): "this option is only valid for purely spatial analyses with the Poisson or Bernoulli models" (Kulldorff 2021). We performed spatio-temporal analysis. In the updated submission we have included in detail how we choose the cluster size in the S3 Appendix.

- Kulldorff M. SaTScanTM User Guide for version 10.0 [Internet]. 2021 [cited 2022 May 30]; Available from: https://www.satscan.org/cgi-bin/satscan/register.pl/SaTScan_Users_Guide.pdf?todo=process_userguide_download

The first sentence of the discussion has already been written in the Introduction. I think the authors could start the discussion by mentioning the main findings of the study

We appreciate your comment. We recap the highlights of the study motivation in the first paragraph of the discussion to make the text more reader-friendly.

line 284: please correct "Ae. aegypti"

Thank you, we corrected it.

In SatScan the authors performed a retrospective analysis of spatiotemporal clusters in Colombia. It would be interesting to see the spatial variations in the temporal trend clusters. The spatial variation in the temporal trends identifies the clusters with growth rates (or decreases) in and out of these clusters, and evaluates whether the difference between them is statistically significant.

This comment is duplicated. It was already addressed above.

The cases of these arboviruses are strictly related to the abundance of the Aedes vector. Would benefit from a short discussion about the climate (temperature, rainfall, humidity, vegetation cover) of the locations where the clusters were identified.

We have included information on the climate of the region where most-likely clusters for dengue, chikungunya, and Zika were identified.

Lines 329-336 (updated version):

> “This region presents a favorable environment and suitable climate for the *Aedes* mosquitoes, with altitude less than 2000 meters above sea level, warm temperatures above 20 ºC, relative humidity below 80%, and rainy seasons that are essential to the life cycle of the mosquitoes [34,58,59]. Cali, where most of the cases in the region concentrate, has a warm and dry climate, with two rainy seasons (from March to May and from September to December). The average annual total precipitation in Cali is 1483 mm, the average maximum temperature is around 30 ºC, and the relative humidity of the air is on average between 70 and 76% [60].”

Is there any control method for Aedes in Colombia? e.g. some insecticide? Maybe discuss a little bit about how this could affect the distribution of cases over time

Despite several efforts for vector control in Colombia, the level of effectiveness of these measures is heterogeneous across the country. Several socio-political and even geographic aspects affect the frequency and quality of the vector control implementation measures in some municipalities. The Colombian government aims at an integrated vector control strategy, as led and indicated by PAHO. The municipal and departmental health secretariats have protocols for vector control activities. These are linked to the national protocols of the Colombian National Institute of Health. The type of biological or chemical control of choice is made upon identification of insecticides resistance, type of entomological indexes to tackle and the opportunity or not of community participatory actions. However, some remote areas or those affected by the armed conflict in Colombia do not necessarily benefit from those vector control strategies. These programs have also been implemented without community participation and without the necessary human resources. Community participation is very important because it’s the community that monitors the appearance of possible breeding sites in their homes every day.

It's very difficult to find correspondence between entomological indexes and the distribution of cases, given that the timing is often off for the entomological surveys which are often only 4 per year in some areas. There are many reasons for the lack of correspondence, including that interventions are only on houses in a block, and sometimes the breeding sites are in the peri-domicile, not inside the house. Also, this information is not often available and if so, heterogeneous in quality or incomplete.

Reviewer #3:

The objectives of the study are clearly articulated and ordered in a logical manner. The study design is appropriate to address the stated objectives. The population is clearly described. I have minor concerns about the methods.

Thank you.

The results follow naturally from the analysis plan and are presented clearly. The figures are best in the .tif format included and seem of sufficient quality.

Thank you.

The conclusions are mostly supported by the data presented and the public health implications are discussed. I think more could be done around the limitations.

Thank you. We would appreciate it if more details on how the limitations can be improved can be provided.

Line 24: (typo) the number of dengue cases reported here do not match the number reported in line 184.<br>Line 96: (typo) shouldn't this be 1,123 municipalities?<br>Line 160, 161: Missing indicator function in equation 6.<br>Line 184: Rewording this sentence so that there aren't 16 numbers in a row might improve readability.<br>Line 186-187: Split this sentence into two. One regarding dengue and one regarding CHIK.<br>Line 218: (typo) ", an e[a]stern pattern"<br>Line 268: (typo) "likely the place of emergenc[e] of new..."

Thank you for indicating these modifications. We fixed the typos.

Lines 160-161 (previous version): The indicator function in equation 6 has a blank space between the parentheses, it is not missing. Following equation 6, it is stated:

> "*I()* is set to 1 when there are more cases than expected, and 0 otherwise."

Line 276-277: why is proximity to Venezuelan border a condition that would favor the introduction of new pathogens? I think additional clarification is necessary here.

Due to the economic crisis in Venezuela sanitary conditions have worsened, allowing the re-emergence of many mosquito-borne diseases. The continuous and uncontrolled mobility of people between Venezuela and Colombia facilitates the transmission of different pathogens around the border. We updated the text to include additional clarification, as suggested.

Lines 306-311 (updated version):

> "It is also close to the Venezuelan border, where there is an important and uncontrolled flow of people given the country's serious economic crisis. The environmental and socioeconomic conditions in this border region facilitate the transmission of infectious diseases including vector-borne diseases [50–52]. If a new pathogen was introduced in the Colombian Atlantic coastline region, its transmission and dispersion would likely be facilitated given these conditions [34]."

Line 286: This sentence could use a bit of reworking for the sake of clarification. What does is mean for a cluster to be "most likely"?

The definition of most likely cluster was included. Lines 322-325 (updated version):

> "For the disease-specific analyses and the multivariate analysis, the most likely cluster, i.e. the one with the strongest evidence of fitting the definition of a cluster, was consistently identified in the south-western region of Colombia, including the Valle del Cauca department and its capital Cali."

Lines 298-307: I think this paragraph needs to be reworked or removed. The point of the paragraph is not coming through clearly. The three cities discussed are not near the border with Ecuador, yet half of the paragraph is about proximity to Ecuador. I can't quite see how these connect.

We rewrote this paragraph to make it clear that it is about the regions where simultaneous clusters were detected.

Lines 340-350 (updated version):

> "Dengue, chikungunya, and Zika simultaneous clusters were identified around the cities of Cali, Ibagué and Neiva, and in western Colombia near the border with Ecuador. Cali, Ibagué and Neiva were part of the most likely clusters for all three diseases separately and simultaneously, and accounted for almost 20% of all cases of dengue, chikungunya, and Zika in the country during the study period. Cali alone contributed 13% of all cases. The western region near the border with Ecuador was previously classified as high-risk for dengue [57]. This region comprises an area with a large proportion of rural population, with limited public, educational, and health services. There is also a very active armed conflict in the region, generated by drug trafficking, further exacerbating health vulnerabilities. Therefore, the important presence of arboviruses observed in this region can be explained by its social and health inequities, in addition to a favorable climate for the presence of the *Aedes* mosquito [34]. "

Thank you for the opportunity to review this work. This research contributes to the understanding of similarities and differences in the spatiotemporal spread of dengue, chikungunya, and Zika across Colombia. The better we can understand these dynamics, the better equipped we hopefully become to disrupt them in the future.<br><br>Below are a handful of comments summarizing revisions or clarifications that I think would strengthen the impact of work. In particular, further clarification around the thresholds used

for cluster detection *and/or* the inclusion of sensitivity analyses varying these thresholds will improve the confidence in the stability of the reported results.

We appreciate the valuable comments and thank you for your careful review. We have prepared a supplementary material (S3 Appendix) with further clarification around the thresholds used for cluster detection. We also explored different maximum cluster sizes and compared the results in this appendix.

Overall, this is informative research that can be even more impactful if it more directly communicates with the findings presented in the existing literature, particularly those constructed from the same (SIVIGILA) data source.

We consider that some of our findings are also discussed in other studies using SIVIGILA data. We have noted this in the discussion and have referenced them accordingly in the text. Here, we are listing some of the mentioned literature:

- Charniga K, Cucunubá ZM, Mercado M, Prieto F, Ospina M, Nouvellet P, et al. Spatial and temporal invasion dynamics of the 2014–2017 Zika and chikungunya epidemics in Colombia. Funk S, editor. PLOS Comput Biol. 2021;17: e1009174. doi:10.1371/journal.pcbi.1009174
- Mora-Salamanca AF, Porras-Ramírez A, De la Hoz Restrepo FP. Estimating the burden of arboviral diseases in Colombia between 2013 and 2016. Int J Infect Dis. 2020;97: 81–89. doi:10.1016/j.ijid.2020.05.051
- Desjardins MR, Whiteman A, Casas I, Delmelle E. Space-time clusters and co-occurrence of chikungunya and dengue fever in Colombia from 2015 to 2016. Acta Trop. 2018;185: 77–85. doi:10.1016/j.actatropica.2018.04.023
- Triana-Vidal LE, Morales-García MA, Arango-Cárdenas MJ, Badiel-Ocampo M, Cuartas DE. Análisis de la distribución espacial y temporal de los virus del Dengue (2006-2017), Zika (2015- 2017) y Chikungunya (2014-2017) en Colombia. Infectio. 2019;23: 352. doi:10.22354/in.v23i4.810
- Lee J-S, Carabali M, Lim JK, Herrera VM, Park I-Y, Villar L, et al. Early warning signal for dengue outbreaks and identification of high risk areas for dengue fever in Colombia using climate and non-climate datasets. BMC Infect Dis. 2017;17: 480. doi:10.1186/s12879-017-2577-4
- Gutierrez-Barbosa H, Medina-Moreno S, Zapata JC, Chua JV. Dengue Infections in Colombia: Epidemiological Trends of a Hyperendemic Country. Trop Med Infect Dis. 2020;5: 156. doi:10.3390/tropicalmed5040156
- Delmelle E, Hagenlocher M, Kienberger S, Casas I. A spatial model of socioeconomic and environmental determinants of dengue fever in Cali, Colombia. Acta Trop. 2016;164: 169–176. doi:10.1016/j.actatropica.2016.08.028
- Desjardins MR, Eastin MD, Paul R, Casas I, Delmelle EM. Space–Time Conditional Autoregressive Modeling to Estimate Neighborhood-Level Risks for Dengue Fever in Cali, Colombia. Am J Trop Med Hyg. 2020. doi:10.4269/ajtmh.20-0080

Methods:
- line 168. A maximum radius of 150 km is set for the space-time clusters. The description

of why this value was chosen is quite vague and non-informative (lines 173-175). I would like to know more about what it means to balance clusters in terms of number and size. How were decisions made about what was "too large" or "too small"? Why is a cluster comprised of a single municipality a bad thing? Previous literature on space-time clusters using municipality data have allowed clusters to be comprised of 1 municipality. Were sensitivity analyses performed for the maximum radius? This information would be useful for future decision-making in future research. I think the evidence for setting the radius could be included in the supplemental material and would be of interest to other researchers.

Thank you for this comment. We have prepared a supplementary material (S3 Appendix) showing the steps taken to define the values used for parameters setting of SaTScan. Different maximum cluster sizes were explored and the results were discussed and compared.

Regarding clusters constituted of one municipality, it is usually a result of setting the “maximum population at risk inside the cluster” too close to the population of the given municipality. In other words, a municipality with a high population concentration and a high disease risk will likely constitute a cluster by itself if the “maximum population at risk inside the cluster” criteria is close to its own population, even if its neighbors also have high risk, because the inclusion of its neighbors would trespass the population maximum size.

- line 176. Why were clusters restricted to a minimum of 100 cumulative cases over the temporal scanning window? What effect does this have on the comparability of dengue versus CHIK and Zika clusters since there are so many fewer cases of CHIK and Zika overall? More discussion of the setting of this minimum threshold and its impact on the results would be informative.

We are now including a supplementary appendix to cover the topics of SaTScan parameters setting. In section 3 of the S3 Appendix we show that using a minimum of 100 cumulative cases inside a cluster does not impact the results compared with using a minimum of 2 cumulative cases.

Results:
- Line 204. In describing the pattern of dispersal from Barranquilla, the text says that there was a southern pattern yet the arrowhead points north.

The yellow arrowhead, corresponding to Barranquilla, points north. This is likely due to edge effects (Moore 1999), given the lack of observations north of Barranquilla and therefore caution should be used in the interpretation. However, observing the black arrowheads we can see a more prominent pattern to the south.

- Moore DA. Spatial diffusion of raccoon rabies in Pennsylvania, USA. Preventive Veterinary Medicine 1999;40(1):19–32.

- Table 1. Related to my comment for lines 176 and 168, I wonder how these cluster results are impacted by the thresholds set on maximum distance and minimum number of cumulative cases per cluster? I recognize the need to set thresholds, but without further information on the validity of the thresholds, it would be useful to know how conclusions change as the thresholds themselves change. It is interesting that the minimum observed number of cases is highest for the disease with the lowest total case count.

We performed the analyses using different parameters settings and organized the results in a supplementary material (S3 Appendix). We hope that now enough information is provided.

Discussion:
- Line 292. Authors note that clusters of Aedes-borne diseases in the south-western region of Colombia have been identified in previous studies. Why weren't they also identified here? Their references (29, 30) analyze the same SIVIGILA data over a similar temporal window at the same level (municipality) with, in the case of reference 29, the same scan statistic. A further discussion of how this work matches and does not match the existing knowledge generated by this data would better situate this work in the context of the literature.

In all four analyses (for each disease in separate and simultaneously), the most likely cluster was indeed detected in the south-western region of Colombia. We mentioned this before but have added more information in the text to make it clearer:

Lines 253-255 (updated version):

> "The most likely clusters for each disease were identified in the same region, in the south-west, including the Valle del Cauca department and its capital Cali."

Lines 281-283 (updated version):

> "The most likely cluster for the multivariate scan analysis was significant for dengue, chikungunya, and Zika (Fig 5A-B), and was also detected in the south-west of Colombia, in the Valle del Cauca department."

Lines 322-329 (updated version):

> "For the disease-specific analyses and the multivariate analysis, the most likely cluster, i.e. the one with the strongest evidence of fitting the definition of a cluster, was consistently identified in the south-western region of Colombia, including the Valle del Cauca department and its capital Cali. This region is known for the presence of Aedes-borne diseases, as it has been described in other studies using the SIVIGILA data [29,30,34,56,57]. Between 2013 and 2016, Valle del Cauca was the department most affected by arboviral diseases in Colombia, accounting for 24.2% of the total disability-adjusted life years (DALYs) of the country and its capital city, Cali, contributed 13% of all arbovirus cases reported during the study period [56]."

Reviewer #4:

Objectives are clearly articulated, however, there is not a clear proposed hypothesis related to the transmission type of disease, seasonality, or geographical regions as initial point of the transmission. The authors can propose a hypothesis (null or alternative) for the temporal trends (seasonality or extreme events and outbreaks), or spatially related to socio-ecological drivers of the transmission (urban determinants of health, human mobility). See:<br>Filho, A.S.N., Murari, T.B., Ferreira, P. et al. A spatio-temporal analysis of dengue spread in a Brazilian dry climate region. Sci Rep 11, 11892 (2021). https://doi.org/10.1038/s41598-021-91306-z<br>Chen et al. 2019. Int J Environ Res Public Health. 2019 Jul; 16(14): 2486. Published online 2019 Jul 12. doi: 10.3390/ijerph16142486

We followed a descriptive approach for these analyses to develop our study objectives, for which the use of hypotheses is precluded. Rather than performing any significance testing procedures to propose or refute any specific hypotheses, we focused on providing a comprehensive picture of the way in which chikungunya and Zika viruses were introduced and describing the presence of disease-specific and overall *Aedes*-borne diseases clusters in the country.

- Yes, the study design seems appropriate to explore the spatio-temporal of high-risk cluster of the diseases either separately or concurrent.<br>- The population of municipalities, as unit of analysis, is described as well the number of cases by epidemiological weeks. However, what is/are the hypotheses that want to be tested?<br>-Yes, municipalities jurisdiction allows a adequate analysis of the whole country. However, the authors need to state clear the hypothesis.

These comments about the hypothesis are addressed in the answer directly above.

- Statistical analysis: I am not an expert in spatial statistics, so I suggest a reviewer with this expertise. However, I wonder about confounding factors such as seasonality in the different regions of Colombia. Are different patterns of rainy and hot temperatures. Considering that dengue, zika, Chikungunya are climate sensitive diseases, something to explore is how the seasonality may be a factor (or not) on the patterns of disease transmission (week number).

Thank you for your comments, indeed in Colombia there are two main rainy seasons and it is generally expected that onset of new outbreaks or epidemic seasons starts a few weeks after their peak, given the likelihood of increased mosquitoes breeding sites. To address the overall time trends and the possibility of high seasonality effects, our analyses were conducted using a dataset in which each row was the count of cases in a given municipality at a given week, which account for the overall time trend. We also assessed the cluster-specific time trends during the study period. For dengue we observed moderate endemicity level with interepidemic periods across years (two to three years) and bimodal trends within

epidemic years. For chikungunya and Zika there were very little or none seasonal trends, their presence was characterized for large outbreaks followed by low endemicity over the study period.

We have included a new figure in the main text with the temporal distribution of cases in each cluster (Fig 4 in the updated version).

- Are there concerns about ethical or regulatory requirements being met?
No, the study met all the requirements of ethics standards.

Thank you.

Yes, the results are linked to the objective of exploring the spatio-temporal clusters for the three diseases in Colombia. The discussion on the geographical sites for high-risk clusters is interesting and supported by the data and references. Yet, is there any additional explanation about the cities connectivity, urbanization trends, human mobility on the period of analysis?

The geography of Colombia makes it possible to think about higher presence across neighboring cities in provinces like Valle del Cauca but less likely in Antioquia, Cauca or Narino, given the natural division created by the Andean mountains. Urbanization and human mobility have increased over the last decades and have an important impact on the spread of infectious diseases.

Line 293: Could be more specific about what are the suitable climate conditions for the diseases.

We have included more details to address this comment.

Lines 329-336 (updated version):

> "This region presents a favorable environment and suitable climate for the *Aedes* mosquitoes, with altitude less than 2000 meters above sea level, warm temperatures above 20 ºC, relative humidity below 80%, and rainy seasons that are essential to the life cycle of the mosquitoes [34,58,59]. Cali, where most of the cases in the region concentrate, has a warm and dry climate, with two rainy seasons (from March to May and from September to December). The average annual total precipitation in Cali is 1483 mm, the average maximum temperature is around 30 ºC, and the relative humidity of the air is on average between 70 and 76% [60]."

- Are the results clearly and completely presented?
The explanation of the temporal cluster analysis considering the peaks of the VBD outbreaks, would need additional discussion: potential relation with extreme years such as El Nino (2015-2016), seasonality (weeks of the year), and interannual variability (2018) compared to other studies on South America region. See:

- Rachel Lowe, Anna M Stewart-Ibarra, Desislava Petrova, Markel García-Díez, Mercy J Borbor-Cordova, Raúl Mejía, Mary Regato, Xavier Rodó. Climate services for health: predicting the evolution of the 2016 dengue season in Machala, Ecuador. The Lancet Planetary Health, Volume 1, Issue 4, July 2017, Pages e142-e151
- Petrova D et al 2020 The 2018–2019 weak El Niño: predicting the risk of a dengue outbreak in Machala, Ecuador Int. J. Climatol. 41 3813–23

We included more discussion about the temporal distribution of the clusters according to the reviewer comments.

Lines 351-355 (updated version):

> "Dengue and Zika clusters were mostly observed between the September of 2015 and the September of 2016. It is possible that during this period the transmission of Aedes-borne diseases was being influenced by the El Niño Southern Oscillation (ENSO). The El Niño is a climate cycle that occurs in the equatorial Pacific Ocean affecting weather patterns, and has been identified as one of the factors increasing dengue transmission in Colombia [64]."

The maps of figure 3 are not easy to understand, a better explanation for:
- The clusters are for all the period (2014-2018), this makes difficult to understand the dynamics of the disease's transmission which is clearly presented in the time series.
- Specially the relative's risk for dengue, zika and chikungunya, individual or in co-occurrence, maybe would be better represented for various points on time (years and weeks) to visualize the temporal dynamics of the disease's transmission.

The maps are depicting the characteristics of the clusters detected using scan statistics, which are, by line: the clusters ranked by likelihood ratio; the year each cluster became "active", i.e., statistically significant; the duration in weeks each cluster was statistically significant; and, finally, the relative risk of each cluster during the time the cluster was statistically significant.

Each cluster has start and end dates, as explained in the methods section (lines 182-184 updated version):

> "Each space-time cluster had its own start and end dates, which were used to calculate the duration in weeks of the cluster, the number of accumulated cases observed in this period, the population within the cluster, and its relative risk."

We have included a new figure (Fig 4 in the updated version) with the temporal distribution of cases inside each cluster to help visualization of the temporal dynamics of the clusters. We have now included supplementary materials (S1-4 Table) to potentially help understanding the cluster characteristics, in particular the time period of each cluster.

It is not feasible to represent the relative risk of the clusters in different time points because the clusters are "active" during a certain time period. Scan statistics provide one estimate of the relative risk for each cluster, that corresponds to the relative risk of the cluster during the time period it is "active".

It is also important to note that although the detection of space-time clusters can give us insights on the temporal dynamics of the diseases, its main objective is to identify where and when the disease is most present.

Yes, partially. The temporal dynamics need to be better explained.

The comment about the temporal dynamics is addressed above.

Need to expand the limitation of the study considering the following ideas:
Vector-borne diseases are complex systems which are driven by natural (climate, environment) and socio-ecological factors. The study just considered the population and number of cases of the diseases individually or in co-occurrence. However, there are many more variables that are relevant in the dynamics of the transmission. Environmental conditions, climate factors, vectors distribution, type of virus circulating, host mobility, and health and policy systems. Some of these limitations may be mentioned in this study.

Thank you for this comment. We have expanded the limitations of the study as suggested (lines 383-386, updated version):

> "Finally, we also acknowledge that Aedes-borne diseases are complex systems, and that there are many other levels necessary to explain disease presence and distribution, including but not limited to environmental conditions, climate, connectivity of municipalities, human mobility, and socioeconomic and demographic characteristics."

- Do the authors discuss how these data can be helpful to advance our understanding of the topic under study?
Yes, with the limitation above mentioned.

Thank you.

- Is public health relevance addressed?
Yes, I suggest that considering issues of global health and climate change.

Thank you for this comment. We have included in the discussion the following (lines 397-401 updated version):

> "It is worth mentioning that the *Aedes aegypti* mosquitoes are increasing their geographic range due to the rising global mean temperature, one of the consequences of climate change, which means regions historically not suitable for these mosquitoes are at risk of future outbreaks [73,74]. The methods described here could also be useful in these settings."

A better explanation on the figure 3 description, and improve the visualization of the risk maps along the time.

This comment is addressed above.

> The study aims to develop new knowledge examining the co-occurrence of all three arboviruses and describing the patterns of introduction including the speed and direction of the spread of chikungunya and Zika in Colombia. The authors propose to apply a cluster analysis to determine unique patterns of spatiotemporal disease risk in Colombia: through disease-specific and multivariate cluster analyses and through estimating the direction and speed of chikungunya and Zika introduction in Colombia.
> Method is promising, however, the inclusion of other factors related with the complex dynamics of the VBD need to be include for future studies. Also, a better visual representation of the dynamics of the cluster may be generated. Considerations of climate variability, change and extremes would need to be included in the limitations of the study, and for future discussions of direction and speed on the region. Global and environmental changes potentially would generate new risk areas for VBD, that would need a better understanding of the dynamics for developing early warning systems for epidemics.

Thank you for your careful review and comments. We hope that we have addressed all your comments to your satisfaction.
